# From Developmental Timing to Clinical Visibility: An Integrative Narrative Review of Sex-Related Neurocognitive Development

**DOI:** 10.3390/children13060725

**Published:** 2026-05-23

**Authors:** Han Gao, Sergey Kiselev, Ningkun Xiao

**Affiliations:** 1 Laboratory for Brain and Neurocognitive Development, Department of Psychology, Institute of Humanities, Ural Federal University, Yekaterinburg 620002, Russia; hgao6408@gmail.com; 2 Department of Immunochemistry, Institute of Chemical Engineering, Ural Federal University, Yekaterinburg 620002, Russia

**Keywords:** sex-related differences, neurocognitive development, gendered experience, developmental timing, phenotypic expression, clinical recognition, autism spectrum disorder, attention-deficit/hyperactivity disorder

## Abstract

Research on sex-related differences in child and adolescent neurocognitive development has often been framed around whether boys and girls differ and which group performs better. This framing is increasingly inadequate because it treats developmental timing, observable phenotypes, and clinical recognition as interchangeable forms of evidence. Drawing on developmental neuroscience, cognitive development, research on gendered experience, and clinical studies of autism spectrum disorder (ASD) and attention-deficit/hyperactivity disorder (ADHD), this integrative narrative review proposes a developmental-visibility framework. The framework interprets sex-related differences across three analytically distinct layers: developmental timing, phenotypic expression, and clinical recognition. Developmental timing refers to age-related trajectories, pubertal coupling, maturation tempo, and variability. Phenotypic expression refers to how developmental differences may appear in language, executive function, emotion, and social cognition under specific task and measurement conditions. Clinical recognition refers to how informants, referral thresholds, compensatory or camouflaging behavior, and diagnostic tools influence which difficulties are identified. ASD and ADHD illustrate the clinical-recognition layer because less externally disruptive or more compensated presentations may remain underrecognized despite meaningful developmental burden. The framework helps explain why modest average effects, inconsistent behavioral findings, and unequal clinical recognition can coexist. It shifts the field from asking whether sex-related differences exist to asking when, how, and under what social and clinical conditions they become visible.

## 1. Introduction: Moving Beyond Mean-Difference Thinking

Research on sex-related differences in child and adolescent neurocognitive development has often been organized around a deceptively simple question: whether boys and girls differ, and which group performs better. This framing is increasingly inadequate. It collapses developmental timing, task-dependent phenotype and clinical recognition into a single mean-difference question. Yet childhood and adolescence are not periods in which two sharply distinct neurocognitive types simply emerge. They are periods in which brain structure, functional connectivity and behavioral regulation are reorganized, and in which sex-related differences may become salient through differences in timing, tempo and variability rather than through stable differences in ability [1,2,3,4,5]. The more informative question is therefore not only whether sex-related differences can be observed, but when they emerge, how they are expressed and under what conditions they become behaviorally or clinically visible [6,7].

This shift is necessary because the most reproducible evidence does not support a simple model of separated male and female neurocognitive profiles. Structural and functional differences reported in developmental neuroscience are often small, may be reduced after accounting for total brain size, sample heterogeneity and multiple comparisons, and occur within highly overlapping distributions [8,9,10,11,12,13,14]. This does not make sex-related developmental differences unimportant. It changes the level at which they should be interpreted. Across the literature, the clearest pattern is not stable rank-order superiority in one group, but modest average differences embedded in substantial individual variability, puberty-linked change and developmentally specific windows of expression [15,16]. Variability is therefore not merely residual noise around a male–female contrast. It is part of the developmental phenomenon that requires explanation.

Existing reviews have typically addressed only one part of this problem. Some have focused on puberty, hormones and brain maturation, particularly structural development and white matter trajectories [17,18,19]. Others have synthesized sex-related differences in a single phenotypic domain, such as language, emotion recognition, executive function or social cognition [20,21,22]. Still others have examined female phenotypes, camouflaging, referral bias or diagnostic delay in ASD and ADHD [23,24]. These level-specific bodies of work provide the foundation for the present review, but they also leave an interpretive gap. Reviews of pubertal maturation and developmental neuroimaging are strongest for explaining when sex-related differences in brain structure, connectivity, or neurophysiological markers become detectable, but they do not by themselves explain why such differences may or may not appear in language, executive function, emotion, or social-cognitive behavior [17,18,19]. Reviews of behavioral and cognitive phenotypes clarify domain-specific patterns, but they often cannot determine whether observed task differences reflect developmental timing, strategy use, measurement context, or gendered experience [20,21,22]. Clinical reviews of ASD and ADHD, in turn, show how camouflaging, informant effects, referral thresholds, and diagnostic tools shape recognition, but they usually begin at the point where a phenotype is already clinically visible [23,24]. The developmental-visibility framework builds on these influential lines of work by placing them into a layer-specific structure of interpretation: developmental-timing models inform the first layer, phenotype-focused cognitive models inform the second layer, and clinical-recognition models inform the third layer. Its added value is therefore not to replace these models, but to clarify what each model can and cannot explain, and to reduce the risk of treating neural timing, task performance, and diagnostic prevalence as interchangeable evidence.

The contribution of this review is not to provide another catalogue of sex-related differences across cognitive domains. Instead, we propose a developmental-visibility framework that organizes the evidence across three analytically distinct layers: developmental timing, phenotypic expression, and clinical recognition. The framework should be read as a conceptual and hypothesis-generating model rather than as an established causal pathway. Its purpose is to clarify which layer of evidence is being measured and to generate testable questions about how developmental timing, behavioral expression, gendered experience, and assessment systems may interact. ASD and ADHD are used as clinical test cases because they show how subtle or compensated phenotypic differences may become differentially recognized by parents, teachers, clinicians, and diagnostic instruments [25,26] (see Table 1 for details).

## 2. Methods and Scope

### 2.1. Review Approach and Conceptual Scope

This article is an integrative narrative review with structured literature identification. Its purpose is to develop a conceptual account of how sex-related differences become developmentally, behaviorally, and clinically recognizable, rather than to provide an exhaustive evidence map or pooled quantitative synthesis. We therefore combined structured literature identification with conceptual synthesis, drawing on developmental neuroscience, cognitive development, gendered social experience, and clinical neurodevelopmental research.

To improve transparency and reduce selective interpretation, we used structured searches in PubMed and Web of Science, supplemented by backward citation tracking of highly relevant reviews and empirical studies. The search strategy was organized around four evidence domains that map onto the logic of the review. First, we prioritized longitudinal neuroimaging, neurophysiological, and puberty-related studies that examined age-related trajectories, pubertal coupling, maturation tempo, or interindividual variability in brain and neurocognitive development. Second, we considered systematic reviews, meta-analyses, and key empirical studies on sex-related differences in language and communication, executive function, emotion, and social cognition. Third, we included theoretical and empirical work on gendered experience, including studies addressing self-concept, task engagement, evaluative interaction, stereotype salience, and social expectations in family, school, peer, media, and institutional contexts. Fourth, we reviewed clinical studies of autism spectrum disorder (ASD) and attention-deficit/hyperactivity disorder (ADHD) that addressed camouflaging, compensation, referral filtering, informant discrepancies, diagnostic sensitivity, and underrecognition. The full search strings, search dates, and evidence charting table are provided in the Supplementary Material.

In brief, the database searches identified 8889 records from PubMed and 16,865 records from Web of Science. After removal of 4827 duplicates, 20,927 records remained for initial screening. Title and abstract screening identified 528 potentially relevant records, of which 110 were retained after full-text assessment and evidence charting. The final synthesis included 110 unique sources: 57 in Flow A, 17 in Flow B, and 36 in Flow C. These included 76 primary empirical studies and 34 review or synthesis sources. Review-level sources were used to identify relatively stable cross-study patterns and areas of disagreement, whereas primary empirical studies were emphasized when they provided direct developmental, mechanistic, or clinical insight.

Eligibility was guided by predefined conceptual and operational rules. Eligible sources had to address human childhood, adolescence, or transition-aged youth when the developmental process of interest clearly began in childhood or adolescence. Flow A sources were eligible when they addressed sex-related or gender-related differences in developmental timing, pubertal coupling, maturation tempo, developmental trajectories, or interindividual variability in brain or neurocognitive development. Flow B sources were eligible when they addressed sex-related or gender-related differences in language/communication, executive function, emotion or emotion recognition/expression, or social cognition, empathy, or prosocial behavior during development. Flow C sources were eligible when they addressed sex-related or gender-related differences in diagnostic visibility, referral filtering, camouflaging, compensation, underrecognition, or clinically meaningful presentation differences in ASD and/or ADHD. Adult-only studies without child or adolescent developmental relevance, animal-only studies, purely somatic or non-neurodevelopmental studies, broad mental-health or educational papers without a direct neurocognitive or clinical-visibility link, and methods-only papers without substantive developmental relevance were excluded.

Several steps were used to reduce selective citation and overinterpretation. First, the search flows were defined before evidence charting and were mapped onto the conceptual layers of the review. Second, database records were deduplicated before screening. Third, candidate sources were screened at title/abstract and full-text levels using the same operational eligibility rules. Fourth, each included study was charted by source, developmental window, broad design or modality, primary domain, and evidence role. Fifth, primary empirical studies and review-level sources were kept analytically distinct, so that review evidence was not treated as equivalent to direct developmental or clinical evidence. Finally, inconsistent findings were not resolved by selecting one preferred direction of effect; instead, discrepancies were interpreted in relation to the layer being measured—developmental timing, phenotypic expression, or clinical recognition.

Evidence selection was guided by conceptual relevance and evidentiary strength. Review-level sources were used to identify relatively stable cross-study patterns and areas of disagreement, whereas primary empirical studies were emphasized when they provided direct developmental, mechanistic, or clinical insight. Greater interpretive weight was given to studies with clear relevance to childhood or adolescence, longitudinal or puberty-sensitive designs, transparent measurement, larger or more representative samples, or direct relevance to ASD/ADHD recognition pathways. Adult-only studies, animal-only studies, methods-only papers, and studies without clear relevance to child or adolescent neurocognitive development were not central to the synthesis, except where they provided necessary conceptual background.

Inconsistent findings were treated as analytically informative. Rather than resolving disagreement by selecting one direction of effect, we used discrepancies to ask which layer of the framework was being measured. A study of pubertal brain maturation, a study of task performance, and a study of diagnostic referral may all be relevant to sex-related neurocognitive development, but they do not estimate the same phenomenon. Disagreements across studies may therefore arise from differences in developmental window, task design, informant source, gendered context, or clinical ascertainment pathway. This approach allowed us to distinguish evidence about developmental timing, observable phenotype, and clinical recognition without treating them as interchangeable.

The scope of the review is centered on childhood and adolescence, with limited extension into transition-aged youth where needed to interpret pubertal or post-pubertal developmental windows. ASD and ADHD are included as clinical test cases rather than as exhaustive representatives of all neurodevelopmental or psychiatric conditions. They were selected because both conditions are subject to substantial child and adolescent research on sex-related presentation, referral, diagnosis, underrecognition, camouflaging, compensation, informant effects, diagnostic thresholds, and tool sensitivity [23,24,27,28]. Their role is to illustrate the phenotype-to-recognition step, especially in conditions where externally visible behavior, internally effortful compensation, and adult interpretation may diverge. Other neurodevelopmental and child mental health conditions were not excluded because they are unimportant, but because they involve different evidence bases and visibility mechanisms that would require a separate review. Anxiety and mood disorders may involve recognition filters related to internal distress, emotional disclosure, help-seeking, and the interpretation of withdrawal or irritability, whereas learning disorders may involve filters related to classroom demands, literacy or numeracy screening, teacher referral, instructional opportunity, and access to psychoeducational assessment. The framework therefore generalizes best as a set of analytic questions—what developmental process is being measured, how is it expressed, and how does it enter recognition systems—rather than as a single disorder-general pathway.

This condition-specific interpretation is consistent with developmental psychopathology work showing that sex-related patterns and observed diagnostic ratios vary across externalizing, internalizing, and learning-related conditions [29,30].

### 2.2. Evidence Strength and Interpretive Boundaries

Because the review integrates evidence from different methodological traditions, we distinguished among relatively well-supported claims, domain-dependent claims, and more preliminary or hypothesis-generating propositions. This distinction is important because developmental neuroimaging studies, behavioral task studies, social-developmental studies, and clinical-recognition studies do not provide the same kind of evidence.

The strongest evidence concerns developmental timing. Longitudinal, puberty-sensitive, and large-cohort neuroimaging or neurophysiological studies provide converging support for the view that sex-related differences during childhood and adolescence are often expressed through timing, tempo, pubertal coupling, regional maturation, and interindividual variability rather than through fixed differences in global ability [3,4,6,13,16,17]. These claims are therefore treated as relatively well supported. Evidence for phenotypic expression is more domain- and task-dependent. Reviews and empirical studies of language, executive function, emotion, and social cognition suggest that sex-related behavioral differences are usually small, context-sensitive, and shaped by task demands, response format, strategy use, and measurement context [20,21,22]. These claims are treated as moderately supported but not uniform across domains.

The evidence for gendered social shaping is conceptually strong and supported by developmental, educational, and experimental literatures on self-concept, stereotype salience, task engagement, and evaluative context [25,31,32,33,34,35,36,37,38,39,40]. However, fewer studies directly measure gendered experience together with pubertal timing, neurocognitive trajectories, and later clinical recognition in the same longitudinal design. This part of the framework is therefore presented as an empirically grounded but still developing mechanism. Similarly, the clinical-visibility layer is best supported in ASD and ADHD, where studies of camouflaging, compensation, informant effects, referral thresholds, and diagnostic tool sensitivity provide direct evidence that recognition is not equivalent to underlying developmental burden [23,24,27,28]. The full timing–phenotype–recognition sequence should therefore be read as an integrative, hypothesis-generating framework rather than as an established causal pathway already tested in its entirety (see Table 2 for details).

## 3. A Developmental-Visibility Framework

### 3.1. Conceptual Clarification: Sex, Gender, and Gendered Experience

Interpreting sex-related differences in child and adolescent neurocognitive development requires a distinction among three related but non-equivalent concepts: sex, gender, and gendered experience. In developmental and neurobiological research, sex usually refers to biological classification and its associated chromosomal, hormonal, reproductive, and anatomical correlates [41]. Gender refers to social roles, norms, identities, and expectations. Gendered experience refers to the patterned opportunities, feedback, evaluations, constraints, and pressures that children encounter within families, schools, peer groups, media environments, healthcare systems, and broader institutions [26].

This distinction is not merely terminological. It determines whether an observed difference is interpreted as a biological input, a socially shaped phenotype, or an assessment-system output. In many developmental studies, the grouping variable is sex as recorded by investigators, whereas the measured behavior may already reflect gendered experience, task familiarity, self-concept, evaluator expectations, response style, and institutional opportunity structures. For this reason, the present review uses the term “sex-related differences” unless the cited literature supports a more specific biological or gender-based interpretation. This wording keeps the interpretation close to the evidence without assigning stronger causal meaning than the data can support.

The distinction also affects how behavioral findings should be read. Observable differences in language use, executive function, emotional expression, or social cognition are not direct readouts of biological sex alone. They may emerge, strengthen, weaken, or disappear depending on developmental timing, social expectations, task design, measurement context, and observer perspective [11,25]. A difference in developmental timing should therefore not be automatically interpreted as a fixed behavioral difference. Similarly, a socially shaped behavioral difference should not be treated as purely biological. The framework developed below depends on keeping these layers analytically separate while recognizing that they interact across development.

### 3.2. Operationalizing Sex, Gender, and Gendered Experience

A practical implication of this distinction is that future studies should avoid treating a single binary grouping variable as if it captured biological sex, gender identity, and gendered developmental experience at the same time. These constructs can be measured and modeled separately. Sex-related biological context can be operationalized through recorded sex at birth or investigator-recorded sex, together with developmentally relevant indicators such as pubertal stage, pubertal timing, age at menarche where applicable, Tanner staging, or hormonal measures when ethically and practically feasible. These measures are especially important in studies of developmental timing, because chronological age alone may not capture puberty-linked neurodevelopmental change.

Gender can be operationalized through age-appropriate self-report of gender identity, gender-role attitudes, gender expression, or perceived gender norms, while recognizing that the available measures must be developmentally sensitive and culturally appropriate. Gendered experience can be assessed through more proximal developmental exposures, including parent and teacher expectations, peer norms, classroom climate, domain-specific encouragement, stereotype exposure, task familiarity, perceived evaluative pressure, and prior opportunities for practice. In clinical and educational studies, gendered experience may also be reflected in informant expectations, referral thresholds, symptom interpretation, and access to assessment.

Analytically, these constructs should not be automatically collapsed. Studies can model sex-related biological indicators, gender-related self-concept or identity, and gendered experience as distinct predictors, moderators, or mediators depending on the research question. For example, a developmental-timing study may test whether pubertal stage moderates age-related change differently by recorded sex. A cognitive-performance study may test whether gendered self-concept, task familiarity, or stereotype salience mediates or moderates task engagement and performance. A clinical-recognition study may test whether parent or teacher expectations, informant discrepancies, or referral thresholds mediate the association between observed behavior and diagnosis. Across these designs, measurement invariance should be examined where possible so that group differences are not inferred from tools that function differently across sex, gender, or informant groups.

This operational approach keeps interpretation close to measurement. If a study measures only recorded sex, its findings should be described as sex-related group differences rather than as evidence for biological causation or gendered social mechanisms. If a study measures pubertal status, self-concept, stereotype exposure, and informant ratings, it can begin to test how biological timing and gendered experience jointly shape phenotype and recognition. The framework therefore encourages more precise measurement rather than stronger causal claims. This approach is consistent with broader reporting recommendations that sex and gender should be reported and interpreted according to what was actually measured rather than used interchangeably [42,43].

### 3.3. The Three-Layer Model

This model is intended to clarify levels of interpretation, not to imply that the three layers have already been validated as a single causal chain. Few studies directly measure developmental timing, gendered experience, behavioral phenotype, and later clinical recognition within the same longitudinal design. The framework is therefore best understood as a hypothesis-generating structure that identifies what future studies should measure together.

Developmental timing is the starting point of the framework. It refers to age-related trajectories, pubertal coupling, maturation tempo, sensitive windows, and interindividual variability. Evidence from longitudinal neuroimaging, puberty-sensitive studies, and large developmental cohorts suggests that sex-related differences are often most informative when they are interpreted as shifts in developmental pace or observability, rather than as global maturity or ability differences [3,4,6,13,16,17].

Phenotypic expression refers to the behavioral form that developmental variation takes. Language, executive function, emotion, and social cognition are measured through tasks, ratings, and social contexts that differ in demand, familiarity, response format, and observer perspective. For this reason, phenotype-level findings are expected to be more variable than timing-level findings. Similar behavioral performance may also reflect different strategies, levels of effort, or neural implementations.

Clinical visibility is the conceptual endpoint of the model and is operationalized here as clinical recognition. A phenotype becomes clinically relevant only when it is noticed, reported, referred, assessed, and named within real-world systems. Recognition is shaped by parent and teacher reports, referral thresholds, diagnostic prototypes, compensatory behavior, camouflaging, and tool design. ASD and ADHD are useful test cases because both show how symptom form, compensation, informant expectations, and referral pathways can shape who is recognized and when [23,24,27,28].

Gendered social shaping operates across these levels. It may influence self-concept, task engagement, evaluative interaction, stereotype salience, stress responding, symptom reporting, and adult interpretation. For example, gendered expectations may affect whether a child persists in a task, how distress is expressed, whether compensatory social strategies are rewarded, or whether quiet inattention is interpreted as anxiety, low motivation, or neurodevelopmental difficulty.

**Figure 1:**
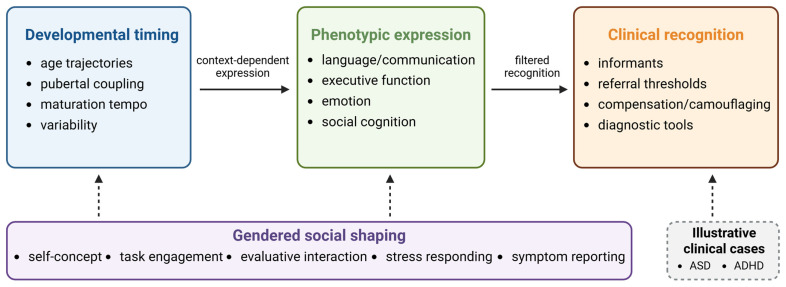
Developmental-visibility framework for sex-related neurocognitive development. The framework distinguishes three interpretive layers: developmental timing, phenotypic expression, and clinical recognition. Gendered social shaping is shown as a cross-cutting process that may influence how developmental differences are expressed and interpreted. ASD and ADHD are used as illustrative clinical cases of the phenotype-to-recognition step rather than as exhaustive representatives of all neurodevelopmental conditions.

This framework helps clarify why findings may appear mixed: studies of developmental timing, task performance, and diagnostic recognition address related but non-identical phenomena. Small average effects can coexist with meaningful developmental timing differences; similar outward performance can coexist with different strategies; and lower diagnosis rates can coexist with substantial developmental burden when recognition depends on compensation, informant mismatch, or referral practices. The following sections use the framework as an interpretive guide: developmental evidence is read in relation to timing, behavioral evidence in relation to phenotype, and ASD/ADHD evidence in relation to recognition (see Figure 1 and Table 3).

## 4. Developmental Timing: Where the Evidence Is Strongest

Among the three levels considered in this review, developmental timing provides the most direct empirical foundation. Across structural MRI, diffusion imaging, functional connectivity, and neurophysiological studies, the most consistent signal is variation in when developmental change accelerates, decelerates, peaks, or becomes more variable [4,6,44]. This evidence is best understood as a shift in developmental windows rather than as a stable difference in ability. A static comparison at one age may suggest a mean difference, whereas a developmental analysis may show that the same difference reflects shifted timing, different slopes, or distinct periods of heightened sensitivity.

Longitudinal and large-scale neuroimaging studies have repeatedly shown that brain development from childhood to early adulthood follows nonlinear trajectories. Early developmental MRI studies reported sex-related differences in the timing of peak values for total brain volume, gray matter, and white matter indices [5,45]. Later work extended this observation across cortical thickness, surface area, cortical folding, limbic morphology, white matter organization, and large-scale functional architecture [46,47,48,49,50]. Taken together, these studies suggest that sex-related differences during development are often differences in pace, sequencing, and regional timing. They indicate when particular neural systems become measurable or reorganized, rather than defining fixed neuroanatomical categories.

A central reason this literature is informative is that it increasingly separates chronological age from pubertal maturation. Puberty is a neuroendocrine transition that overlaps with age but is not reducible to age. Studies that model pubertal stage or pubertal hormones suggest that puberty is associated with changes in cortical thickness, surface area, hippocampal development, white matter microstructure, and resting-state connectivity, with some associations differing by sex [6,17,51,52,53,54,55,56,57]. Age-only models can therefore obscure the mechanism by treating children at the same chronological age as developmentally equivalent. Puberty-sensitive models instead clarify when sex-related differences are most likely to become detectable.

White matter and network-level studies support the same timing-based interpretation. Diffusion imaging studies have reported sex-related patterns in white matter architecture and tract maturation during late childhood and adolescence, while puberty and hormones appear to contribute to microstructural change in ways that are not captured by age alone [17,49,52,58,59,60,61]. Functional studies further indicate that association networks, reward-related processing, and spontaneous cortical dynamics may show sex-related developmental organization or timing effects [50,62,63,64,65,66,67]. The common implication is that observability depends on system, marker, and window. A difference may appear in one neural system or developmental period and be absent in another, not because the evidence is incoherent, but because the timing of measurement matters.

A further development in this literature is the move from group means to variability. Developmental datasets show substantial within-sex heterogeneity, and some studies suggest sex-related differences in variance structure or interindividual variability in structural development [13,15,16]. This shift from average difference to developmental dispersion is important for both behavioral and clinical interpretation. Variability may influence which children fall into more vulnerable developmental tails, which phenotypes become detectable, and which difficulties later enter referral systems. Later behavioral or clinical differences may therefore emerge from the interaction of subtle timing shifts with heterogeneous developmental pathways, rather than from large average differences between groups.

The timing-based account also helps explain why some clinically relevant differences become more apparent during adolescence. Pubertal timing and tempo have been linked to mental health risk and may differ in neurodevelopmental conditions, including ASD, where studies have examined altered pubertal timing or progression with possible implications for later burden and recognition [68,69]. Early hormonal windows, such as mini-puberty, have also been investigated as possible contributors to later sex-related neurodevelopmental variation, although downstream associations remain uncertain and should be interpreted cautiously [70]. These findings do not imply a direct path from hormones to behavior. They indicate that endocrine and maturational timing may change the conditions under which later phenotypes become more or less detectable.

Developmental timing should therefore be understood as a probabilistic constraint on phenotypic expression rather than as a direct determinant of ability. This timing-based interpretation provides the bridge to the phenotype literature, where developmental signals are measured through tasks, strategies, observers, and social contexts.

## 5. Phenotypic Expression: Why Behavioral Findings Are Inconsistent

Phenotypic evidence is less uniform than evidence on developmental timing because behavior is measured through tasks, observers, and social contexts. Across language, executive function, emotion, and social cognition, reported sex-related differences are usually small, sensitive to construct definition, and moderated by age window, task format, informant source, and evaluative context [71,72,73]. At the phenotype level, the core problem is translation: the same developmental condition may produce different behavioral signals depending on task format, strategy availability, motivational context, and observer sensitivity. Phenotypic inconsistency is therefore analytically informative. It helps identify where developmental variation becomes measurable, muted, compensated, or socially interpreted.

Some behavioral domains do show reproducible average differences, but their interpretation depends on age, task format, informant source, and measurement context. A finding may be robust as a task effect, developmentally limited as an age-window effect, socially shaped as a response-style effect, or clinically relevant as a recognition effect. These possibilities should not be collapsed into a single claim about stable ability.

### 5.1. Language and Communication: Small Early Differences, Weak Stability

Language and communication are often treated as domains in which girls show an early advantage. The available evidence supports a more specific interpretation. Normative and meta-analytic studies suggest that girls may show small early advantages in some aspects of communication and language use, particularly during infancy and early childhood, but these effects explain limited variance and are moderated by age, context, interaction partner, and measurement approach [71,74]. These findings are more informative as developmental signals than as evidence for a stable language advantage.

The interpretation also depends on which aspect of language is being measured. Expressive vocabulary, receptive language, grammar, reading, pragmatic communication, conversational behavior, and parent-reported communication do not capture the same phenotype. A small difference in early expressive language does not necessarily imply a durable difference in pragmatic competence, social communication, or adaptive language use. The link between language-related neural development and observed language performance is also indirect. A related review concluded that evidence for sex-related differences in language-relevant brain structure and function is limited and inconsistent [21]. Even when neural differences are reported, they do not map straightforwardly onto language outcomes.

This distinction matters because language can alter recognition pathways without reflecting broad neurocognitive superiority. A child who appears verbally competent in structured settings may still experience pragmatic, social-communication, or adaptive difficulties that are not immediately detected. This point becomes relevant in ASD, where superficially fluent communication can complicate recognition when social difficulties are compensated, scripted, or masked.

### 5.2. Executive Function and Cognitive Control: Component-Specific Rather than Global Differences

Executive function provides an especially clear example of why phenotype should not be treated as a unitary outcome. The term covers multiple control processes, including inhibition, working memory, cognitive flexibility, sustained attention, planning, reward sensitivity, and delay-related decision-making. These processes mature at different rates and are commonly assessed using non-equivalent tasks. As a result, sex-related findings in executive function are difficult to summarize as a single pattern.

Longitudinal and large-sample studies suggest that some executive subcomponents may show different developmental trajectories in boys and girls, including differences in baseline performance, rate of change, or within-person variability [75,76]. However, these patterns do not support a global claim that one sex has better executive control. Evidence syntheses are consistent with this caution. Some meta-analytic findings suggest sex-related differences in specific constructs, such as inhibitory control or delay-related decision-making, whereas broader claims about executive function remain inconsistent [77,78].

A more useful interpretation is component-specific. Inhibition may shape overt behavioral control; working memory may affect classroom task management; sustained attention may influence academic persistence; cognitive flexibility may affect adaptation to changing task rules; and reward sensitivity or delay-related decision-making may alter risk-taking, motivation, and response to feedback. These components differ in developmental timing and in how easily adults notice them. Neuroimaging evidence reinforces the need for this task-specific interpretation. Reviews of task-related functional imaging indicate that sex-related differences in cognitive and affective tasks depend on task demands, analysis choices, and sample characteristics [72,73]. Similar behavioral performance may also be supported by different strategies or neural implementations, which means that outward task scores may underestimate differences in effort, strategy, compensatory control, or neural recruitment.

Executive-function findings are clinically relevant because different profiles of regulation, attention, and behavioral salience may draw different levels of adult concern. In ADHD, externally disruptive hyperactivity may trigger referral more readily than inattentive, internally effortful, or compensated presentations. The clinical significance of executive-function findings therefore lies in how specific regulatory profiles enter recognition pathways, rather than in any global ranking of control ability.

### 5.3. Emotion and Stress-Related Expression: Expression Style Rather than Emotional Ability

Emotion-related findings are somewhat more consistent than many other phenotypic domains, but they still require careful interpretation. Meta-analytic evidence suggests small sex-related differences in children’s emotional expression, with girls tending to show more positive and internalizing emotional expression and boys tending to show more externalizing emotional expression [20]. A separate meta-analysis found a small female advantage in recognizing non-verbal emotional displays, although effect sizes varied by emotion type, modality, and age [22]. These findings suggest differences in expression and recognition patterns, not a general difference in emotional capacity.

This literature is best separated into at least four related processes: emotion recognition, emotion expression, emotion regulation, and stress reactivity. Emotion recognition concerns how children identify affective cues. Emotion expression concerns how feelings are displayed. Emotion regulation concerns how affective states are modulated across contexts. Stress reactivity concerns physiological and behavioral responses to challenge or evaluation. These processes can diverge. A child may recognize emotions accurately but suppress expression, regulate effectively in structured settings but not under peer stress, or experience high internal distress with little overt disruption.

Developmental timing is especially relevant in this domain. Adolescence brings changes in affective salience, social evaluation, stress reactivity, and emotion-regulation demands. Pubertal maturation may interact with neural systems involved in emotion processing, including limbic and prefrontal networks, in ways that vary across individuals and contexts [79,80]. Pubertal timing has also been associated with risk for internalizing and externalizing problems, although sex-specific pathways are heterogeneous and should not be reduced to a single mechanism [81].

The mechanism linking emotion to recognition is therefore expression-dependent. Emotional burden may appear as overt dysregulation, withdrawal, anxiety, irritability, somatic complaints, overcontrol, or compensatory effort, depending on developmental stage and social context. Because internal distress and external behavior are not equally likely to be noticed or referred, similar developmental burdens may produce different recognition outcomes.

### 5.4. Social Cognition and Empathy: Response Style, Norms, and Compensation

Social cognition and empathy are often discussed as domains of sex-related difference, but the interpretation of this literature depends strongly on measurement. Longitudinal and review-level evidence suggests that girls may show higher average levels on some measures of empathy, perspective-taking, or prosocial behavior, and that some differences may become more apparent during adolescence [82,83,84]. Meta-analytic work on prosocial behavior also suggests that effect sizes vary by subtype of prosocial behavior, age, and context [85]. These patterns are meaningful, but they are not independent of task design, self-report norms, or social expectations.

This domain is particularly sensitive to response style and cultural meaning. Self-reported empathy, observed helping behavior, parent-rated prosociality, and laboratory social-cognitive tasks do not measure the same construct. Some measures capture motivation to respond, others capture emotion recognition, perspective-taking, social knowledge, conformity to expectations, or willingness to report socially valued traits. Sex-related differences in these outcomes may therefore reflect both developmental processes and gendered expectations about relational behavior, emotional attunement, and social responsibility.

The link with camouflaging clarifies why social cognition should not be interpreted only as a level of ability. In some children, social knowledge may support adaptive interaction; in others, it may support monitoring, imitation, scripting, or compensatory performance that reduces outward atypicality while increasing effort. This is especially relevant in ASD. Girls may develop social strategies that reduce overt atypicality in structured or familiar settings while leaving underlying social-cognitive effort, exhaustion, or uncertainty less apparent. Studies of female autistic phenotype and camouflaging suggest that outward social adaptation can alter recognition pathways, especially when assessment tools or referral expectations are calibrated to more externally visible presentations [27,86,87,88,89]. These findings do not imply that social cognition is uniformly stronger in girls. They suggest that social performance, compensatory strategy, and clinical recognition can become dissociated.

Taken together, the phenotype literature shows why behavioral findings are often uneven across tasks and domains. Language, executive function, emotion, and social cognition are measured through different task formats, response demands, social meanings, and informant perspectives. These conditions determine which developmental signals become measurable and which remain compensated, muted, or interpreted differently. The next section considers the social mechanisms that shape this translation in everyday contexts.

## 6. Gendered Social Shaping: Why Timing Does Not Become a Phenotype Directly

Gendered social shaping helps specify how developmental variation is expressed in everyday contexts. Children develop in families, classrooms, peer groups, media environments, clinical encounters, and institutional settings. These contexts influence what children practice, what they believe they are good at, how they respond under evaluation, how distress is expressed, and how adults interpret behavior. Gendered experience therefore provides a mechanism by which developmental capacity becomes task engagement, response style, symptom expression, or adult concern.

This mechanism does not replace developmental biology. It conditions how biological and developmental signals are expressed. A difference in pubertal timing, maturation tempo, or neural variability may have different outward consequences depending on opportunities, expectations, confidence, peer context, and adult observers. For this reason, gendered social shaping is treated here as a cross-cutting process rather than as a fourth layer parallel to developmental timing, phenotypic expression, and clinical visibility.

This cross-cutting role is supported by newer evidence that extends classic gender-socialization theories. A preregistered meta-analytic review of 98 studies found that children’s gender stereotypes about STEM and verbal abilities vary by domain and developmental period, suggesting that gendered beliefs are not uniform but become organized around specific tasks and cultural meanings [33]. Recent work also shows that children’s stereotypes and motivation can diverge across STEM fields, with computer science and engineering carrying stronger boy-favoring stereotypes than mathematics or general science in some samples [32]. At the classroom level, students’, parents’, and peers’ gender stereotypes have been associated with adolescents’ self-concept, interest, and anxiety in mathematics, indicating that gendered expectations may operate through motivational and affective pathways rather than through ability alone [31]. Together, these studies support the view that gendered experience can shape whether a developmental capacity becomes engagement, confidence, avoidance, anxiety, or measured performance.

To make these links explicit, gendered social shaping can be understood as a set of measurable pathways rather than as a general background context. Stereotype salience may influence whether a task is experienced as identity-congruent, threatening, or evaluative, with measurable consequences for task engagement, response latency, error monitoring, persistence, and performance variability. Evaluative interaction may affect how children regulate behavior in front of parents, teachers, peers, researchers, or clinicians, and may therefore be reflected in cautious responding, disengagement, overcompensation, or changes in observed executive-control and emotion-regulation behavior. Gendered self-concept may shape domain-specific confidence, perceived competence, strategy choice, and willingness to attempt difficult items. Adult expectations may influence informant ratings, referral concern, and whether the same behavior is interpreted as anxiety, low motivation, immaturity, impulsivity, or neurodevelopmental difficulty. These pathways are measurable through task process indices, repeated assessments, self-concept and motivation scales, observer ratings, parent/teacher reports, and experimental or quasi-experimental manipulation of evaluative context.

This clarification also helps define what the framework does and does not claim. The argument is not that social mechanisms create all sex-related differences in neurocognitive development. Rather, gendered experience may alter how developmental capacities are expressed during assessment and how expressed behaviors are interpreted by observers. Thus, the same developmental capacity may lead to different measured outcomes depending on task familiarity, stereotype salience, perceived evaluative pressure, and informant expectations. Conversely, similar task scores may reflect different levels of effort, strategy use, stress, or compensation. These process-level measures are therefore essential for connecting social mechanisms to neurocognitive and behavioral outcomes (see Table 4 for details).

### 6.1. Self-Concept and Task Engagement

One pathway through which gendered experience may shape phenotype is self-concept. Social cognitive accounts of gender development emphasize that children learn gendered expectations through observation, reinforcement, modeling, and self-regulation. Developmental intergroup theory further suggests that children attend to social categories that are made salient in their environments; when gender is repeatedly used to organize activities, expectations, or evaluation, it can become a meaningful dimension of self-understanding [25].

Empirical studies support this mechanism. Children can acquire gendered beliefs about domains such as mathematics, intellectual ability, and social behavior early in development, and these beliefs may influence interest, confidence, and engagement before they are reflected in stable achievement differences [34,35,36,37,38]. These findings are relevant to neurocognitive development because task performance depends partly on whether children approach a task as familiar, valued, threatening, effortful, or identity-congruent.

Stereotype-relevant cues may alter self-concept; self-concept can shape confidence, error monitoring, and willingness to persist; these processes then affect measured performance. A child’s underlying cognitive resources may not be fully expressed if a task is experienced as identity-incongruent or evaluatively threatening. Conversely, encouragement, practice, and confidence may increase performance in contexts where the child feels competent. This pathway helps explain why small developmental differences may appear stronger, weaker, or absent across tasks and settings.

### 6.2. Evaluative Interaction and Stereotype Salience

A second pathway involves evaluative interaction. Children do not perform cognitive, emotional, or social tasks in neutral environments. They are observed by parents, teachers, peers, researchers, and clinicians, and these observers bring expectations about maturity, competence, sociability, emotional control, and classroom behavior. Such expectations may influence how children behave during assessment and how their behavior is interpreted.

Experimental and school-based studies suggest that increasing the salience of gender categories can strengthen stereotype-consistent beliefs and peer differentiation [40]. Related work on early stereotypes about ability and domain belonging indicates that children’s interests and self-evaluations may be shaped by gendered messages before formal educational tracking occurs [34,35,36]. Stereotype threat and evaluative pressure may also affect performance in specific contexts, although these effects are likely to vary by age, domain, cultural setting, and task design [39].

This pathway is especially relevant to measurement because evaluative settings can change the process being measured. A child may use a more cautious strategy, monitor errors more closely, disengage earlier, or overcompensate when a task carries gendered meaning. Adult observers may also interpret the same behavior differently depending on whether it appears as quiet inattention, anxious overcontrol, defiance, immaturity, impulsivity, or lack of effort. Thus, school and assessment expectations can move behavior along different interpretive routes: inattention may be read as low motivation, anxiety, or neurodevelopmental difficulty depending on the observer and context.

This mechanism is particularly relevant for executive function, emotion, and social cognition, which are often measured through observed regulation, social interpretation, self-report, or adult ratings. Apparent sex-related differences may partly reflect how children respond to evaluative settings and how adults interpret behavior in those settings. Evaluative interaction can therefore change both the behavior that is expressed and the likelihood that it is treated as meaningful.

### 6.3. Stress Response and Symptom Reporting

A third pathway involves stress response and symptom reporting. Emotion-related differences in childhood and adolescence are often small but context-sensitive, and they vary by age, interaction partner, and social setting [20,22]. Puberty may further alter affective salience, stress reactivity, and social-evaluative sensitivity, creating windows in which internalizing and externalizing pathways become more differentiated across individuals [79,80,81]. These developmental processes may be shaped by expectations about how boys and girls should express distress, seek help, or regulate behavior.

A related emotional and clinical pathway is that gendered emotional norms may influence whether distress is expressed as internalizing symptoms, externalizing behavior, overcontrol, somatic complaints, irritability, or help-seeking. These forms of expression then shape adult concern and referral. Recent meta-analytic evidence on parental emotion socialization indicates that supportive and nonsupportive parental responses are associated with children’s internalizing problems, although effects vary in size and do not imply a simple parent-to-child pathway [90]. This evidence strengthens the argument that emotional expression and symptom reporting are socially embedded. Clinical systems often respond more readily to behaviors that disrupt others than to symptoms that are internally burdensome but externally less apparent. Symptom form can therefore influence whether difficulty becomes legible as developmental, emotional, behavioral, or disciplinary.

Stress response and symptom reporting connect emotional phenotype with recognition. A child may experience meaningful difficulty, but that difficulty may remain less apparent if it is expressed through internal effort, compensation, or socially acceptable overcontrol. By contrast, disruptive symptoms may trigger earlier concern, referral, or diagnosis. Gendered expectations can therefore influence not only how distress is expressed, but also which forms of distress become legible to adults.

Together, these mechanisms show how social context can amplify, dampen, or redirect subtle developmental differences as they become behavior. Self-concept, evaluative interaction, and symptom reporting do not operate outside neurocognitive development; they help determine how developmental signals are expressed and interpreted. The clinical question is what happens next when these already shaped presentations enter assessment and referral systems. Direct longitudinal studies that measure gendered experience, pubertal timing, neurocognitive phenotype, and clinical recognition within the same design remain rare. For this reason, gendered social shaping should be read as a theoretically and empirically supported bridge, rather than as a fully specified causal pathway.

## 7. Clinical Visibility: ASD and ADHD as Test Cases

Clinical recognition provides a practical illustration of the proposed framework. Developmental and behavioral differences enter assessment systems through parent and teacher reports, school expectations, referral thresholds, diagnostic prototypes, compensatory behavior, and tool design. ASD and ADHD are used here as illustrative clinical cases because both have developed research on underrecognition, camouflaging, compensation, informant discrepancy, referral filtering, and delayed diagnosis. Their value is analytic rather than representative: they show how symptom form and observer expectations can shape whether a phenotype becomes recognizable. In ASD, this problem is especially evident in camouflaging, compensation, social-language presentation, and parent–teacher discrepancies. In ADHD, it is especially evident in symptom presentation, diagnostic thresholds, referral pathways, and the transition from symptoms to diagnosis or treatment. Other conditions, including developmental language disorder, dyslexia, anxiety, and mood disorders, may involve different recognition pathways and require separate evidence bases.

### 7.1. ASD: The Phenotype Can Remain Socially Less Recognizable

The ASD literature increasingly suggests that autistic girls and women may show presentations that are less well captured by diagnostic expectations derived largely from male-dominated clinical samples. Systematic and review-level evidence indicates that sex- or gender-related differences in autistic social interaction and communication are often construct-specific, with some studies finding relatively stronger performance among autistic females on narrow social-communication measures [89]. Such findings require careful interpretation. They do not show an absence of difficulty; they suggest that some difficulties may be expressed in ways that are less readily detected by standard tools or observers.

Camouflaging and compensation are central to this recognition problem, but the pattern is heterogeneous. Some autistic girls and women may use imitation, rehearsed conversational strategies, social scripting, or increased monitoring of their own behavior to reduce outward atypicality in familiar or structured settings [23,27,86]. Others may not camouflage successfully, may camouflage only in specific settings, or may show difficulties that are recognized through anxiety, exhaustion, peer problems, or emotional dysregulation. The relevant point is not that autistic girls uniformly camouflage, but that compensation can weaken the link between developmental burden and outward detectability.

Language and pragmatic presentation may also affect recognition. Sex-linked social-language features have been proposed as one reason why autistic girls can appear more socially typical during assessment or school observation, despite ongoing social-cognitive effort and vulnerability [87,88,91]. Diagnostic tools and referral pathways may further shape recognition. If behavioral examples, rating thresholds, or clinician expectations are calibrated to more externally visible or male-typical presentations, compensated presentations may be referred later or may require greater co-occurring distress before recognition occurs [27,92,93]. Informant discrepancies can also matter: a child may appear socially engaged in one setting but exhausted, anxious, or dysregulated in another.

For the proposed model, ASD illustrates how a socially shaped and partly compensated phenotype can remain sufficiently functional in some settings to escape standard detection while still carrying substantial internal burden. Its relevance lies in separating outward detectability from developmental need.

### 7.2. ADHD: Recognition Depends on Symptom Form and Observer Expectations

ADHD illustrates a related but distinct recognition problem. Review and meta-analytic evidence suggests that boys are more likely to show overt hyperactivity, impulsivity, and disruptive classroom behavior, whereas girls may more often present with inattentive symptoms, internalizing difficulties, social problems, or compensatory effort [24,28]. These are group-level tendencies, not categorical profiles. They vary across development, setting, symptom domain, and referral pathway. Their value for the present framework is that they show how symptom form can influence whether a difficulty is noticed and referred.

Referral is particularly sensitive to observer expectations. Teacher and parent reports do not always converge, and referral decisions may vary according to both child gender and symptom type [94,95]. Girls with substantial ADHD symptoms may be less likely to receive diagnosis or treatment if their difficulties are less disruptive or are interpreted as anxiety, immaturity, low motivation, or personality style rather than neurodevelopmental impairment [96,97,98]. Clinical samples may therefore overrepresent children whose symptoms are most apparent to adults and underrepresent those whose difficulties are internally burdensome but externally less disruptive. Recent evidence also suggests that teacher reports and referral judgments can vary across cultural and educational contexts, reinforcing the need to interpret ADHD recognition as an informant- and system-dependent process rather than a simple reflection of symptom presence [99].

Developmental trajectories further complicate this picture. Community-based studies suggest that girls’ ADHD-related difficulties can remain clinically consequential even when they are not captured early by clinic-driven systems [96,100]. Delayed recognition may have downstream implications for treatment access, academic support, emotional burden, and self-understanding. The mechanism is therefore not simply a sex difference in severity but a recognition pathway shaped by symptom expression, adult interpretation, and referral context.

For the proposed model, ADHD illustrates how symptom form and informant expectations jointly shape recognition. Observed sex ratios should therefore be interpreted in relation to ascertainment pathways, not as direct estimates of true prevalence or severity.

### 7.3. Clinical Recognition as a Filtered Endpoint

ASD and ADHD research make the recognition step concrete. A developmental study may detect differences in timing or variability; a behavioral study may find small, task-dependent effects; a clinical study may report marked sex ratios in diagnosis. These outcomes should not be collapsed into a single interpretation. They correspond to different points in the path from developmental process to observed behavior to clinical recognition.

This distinction has practical consequences. Population-based and community-based samples are needed alongside referral samples. Measurement invariance and informant effects should be tested more routinely, especially in social cognition, emotion, and clinical assessment. For clinical practice, lower recognition should not be taken as evidence of lower burden. Assessment systems may need broader behavioral examples, explicit attention to compensation and camouflaging, and greater sensitivity to internally effortful or less disruptive presentations.

This interpretation should not be extended uncritically from ASD and ADHD to all neurodevelopmental or child mental health conditions. Other conditions may involve different recognition filters. Anxiety and mood disorders may be missed when distress is internalized, normalized, or attributed to personality or developmental stress. Learning disorders may be missed or delayed when academic difficulty is attributed to effort, instruction, language exposure, or classroom adjustment rather than to a specific learning profile. The developmental-visibility framework can therefore guide comparative questions across conditions, but each condition requires its own evidence base and measurement model.

## 8. Testable Propositions and Methodological Implications

The proposed framework is useful only if it generates testable questions. At present, developmental timing, behavioral phenotype, gendered experience, and clinical recognition are often studied separately. Longitudinal neuroimaging studies clarify timing; task-based studies capture behavioral expression; and ASD/ADHD research shows how assessment systems shape recognition. The next step is to test the transitions among these components.

We organize the research agenda into two groups. The first concerns developmental mechanisms: how timing becomes phenotype, how gendered experience moderates this translation, and whether similar performance reflects similar underlying processes. The second concerns measurement and recognition: how systems detect phenotype and whether current tools measure constructs equivalently across groups and informants.

### 8.1. Core Developmental Propositions

#### 8.1.1. Proposition 1: The Timing-to-Phenotype Hypothesis

Sex-related differences in neurocognitive development are most informative when developmental timing is linked to phenotypic windows. Pubertal timing, maturation tempo, and interindividual variability may predict when differences in language, executive function, emotion, or social cognition become measurable, rather than whether one group shows a stable advantage across development. Longitudinal and puberty-sensitive studies suggest that brain maturation is shaped by age, puberty, hormones, and individual variability in ways that cannot be captured by age alone [6,13,16,17,19]. Future studies should therefore model chronological age and pubertal status together, test nonlinear trajectories, and estimate variability rather than focusing only on mean differences.

#### 8.1.2. Proposition 2: The Social-Shaping Moderation Hypothesis

Gendered experience may moderate the translation from developmental timing to observed phenotype. Self-concept, stereotype salience, task engagement, family and school expectations, and evaluative interaction may shape whether a developmental capacity is expressed, suppressed, compensated, or interpreted as difficulty. This proposition is consistent with social cognitive and developmental intergroup accounts of gender development [25] and with newer evidence linking children’s gendered ability beliefs and classroom stereotypes to self-concept, motivation, and anxiety [31,32,33]. Future research should measure gendered experience directly rather than using recorded sex as a proxy for all sex- and gender-related processes.

#### 8.1.3. Proposition 3: The Strategy-Equivalence Hypothesis

Similar behavioral performance may be supported by different strategies, levels of effort, or neural implementations. This proposition prevents similar task scores from being interpreted as evidence that the underlying developmental process is the same. Future studies should combine behavioral outcomes with process-sensitive indicators such as reaction-time dynamics, error patterns, strategy reports, eye tracking, computational modeling, physiological stress markers, neuroimaging, or repeated within-person assessments. The goal is to distinguish shared performance from shared process.

### 8.2. Measurement and Clinical Propositions

#### 8.2.1. Proposition 4: The Recognition-Filter Hypothesis

The same phenotypic burden may lead to different probabilities of clinical recognition depending on informant perception, referral thresholds, diagnostic tool design, and compensatory behavior. ASD and ADHD provide clear examples. In ASD, camouflaging, linguistic compensation, informant discrepancies, and diagnostic expectations may reduce recognition or delay identification in some children [23,27,86,87,93]. In ADHD, symptom form and observer expectations may influence whether difficulties are noticed and referred, particularly when symptoms are inattentive, internally effortful, or less disruptive [24,28]. Future studies should compare community-based, school-based, and clinic-referred samples and use multi-informant designs to identify which difficulties become recognizable in which settings.

#### 8.2.2. Proposition 5: The Measurement-Invariance Hypothesis

Tasks, rating scales, and diagnostic tools should not be assumed to measure the same construct equivalently across sex, gender, culture, and informant source. At the phenotype level, language, emotion, executive function, and social cognition tasks vary in familiarity, social meaning, and response demands. At the clinical level, symptom checklists and diagnostic interviews may be more sensitive to externally disruptive behaviors than to compensated, internally effortful, or context-dependent difficulties. Future research should routinely evaluate measurement invariance and evaluator effects, especially in domains with high observer dependence. For ASD and ADHD, clinical tools may need broader behavioral examples, explicit assessment of compensation and camouflaging, and greater sensitivity to internally burdensome but externally less disruptive presentations.

### 8.3. Methodological Priorities

These propositions point to several methodological priorities. Developmental studies should integrate age, puberty, and individual variability within longitudinal or accelerated longitudinal designs. Sex, gender, and gendered experience should be separated conceptually and operationally rather than collapsed into a single binary grouping variable [26,41]. Phenotype-level studies should test whether similar behavioral outcomes reflect similar processes. Clinical studies should examine how informant discrepancies, diagnostic tools, and compensatory strategies shape recognition. Cross-cultural and institutional contexts also require closer attention, because school systems, healthcare pathways, and gender-norm environments may influence both expression and recognition [33,99,101].

The most informative future designs will connect developmental timing, social experience, behavioral process, and recognition within the same longitudinal or multi-informant framework.

### 8.4. Practical Implications for Clinical Assessment and Study Design

The framework has several practical implications for assessment. For clinicians, the main implication is that lower external visibility should not be equated with lower developmental burden. In ASD and ADHD assessment, this means that clinicians should ask not only whether a behavior is observable to adults, but also how much effort is required to maintain outward functioning, whether the child uses compensatory or camouflaging strategies, and whether difficulties are expressed differently across home, school, peer, and assessment settings. Multi-informant assessment is especially important because parent, teacher, and self-reports may capture different parts of the phenotype rather than simply agreeing or disagreeing. A quiet or academically compliant child with substantial internal effort, social fatigue, anxiety, or attentional strain should therefore not be assumed to be low risk solely because disruptive behavior is limited. This point is consistent with broader evidence that cross-informant discrepancies in child assessment can be clinically informative rather than merely measurement error [102].

For researchers, the framework suggests that studies should be designed to separate developmental timing, phenotypic expression, gendered experience, and recognition wherever possible. A developmental study could measure chronological age, pubertal stage, and repeated neurocognitive outcomes to test whether sex-related differences reflect timing or trajectory rather than static group differences. A cognitive study could include process-sensitive measures such as reaction time, error patterns, strategy reports, task engagement, and physiological or neural indicators to test whether similar performance reflects similar underlying processes. A clinical-recognition study could compare community, school, and clinic samples; test measurement invariance across sex or gender groups; and examine whether informant expectations, referral thresholds, or camouflaging explain who receives diagnosis or support. These examples illustrate how the framework can be translated into testable designs rather than remaining only a conceptual account (see Table 5 for details).

### 8.5. Limitations and Future Directions

A further limitation is that the full developmental-visibility sequence has not yet been tested directly as a longitudinal causal pathway. The framework integrates evidence across the literature, but most studies measure only one or two layers at a time. For example, puberty-sensitive neuroimaging studies can clarify developmental timing but usually do not follow children into diagnostic-recognition systems; clinical studies of camouflaging or referral bias can clarify recognition but usually do not reconstruct earlier neurodevelopmental timing. Future work should therefore test the framework prospectively by measuring age, pubertal status, neurocognitive trajectories, gendered experience, task process indicators, informant reports, and referral or diagnostic outcomes within the same design. Until such evidence is available, the framework should be interpreted as a conceptual and hypothesis-generating model.

## 9. Conclusions

Current evidence does not support two sharply distinct developmental neurocognitive profiles in children and adolescents. A more defensible interpretation is that sex-related differences are usually modest, developmentally contingent, and shaped by the conditions under which they are measured and recognized.

The developmental-visibility framework shifts interpretation away from asking only whether boys and girls differ and toward a more precise set of questions: which developmental layer is being measured, under what task or social conditions a phenotype becomes visible, and how recognition systems shape who is identified. Its value lies in separating developmental timing, phenotypic expression, gendered experience, and clinical recognition so that future studies can test their links directly rather than treating them as interchangeable evidence.

Clinically, the framework cautions against equating lower recognition with lower burden. In ASD and ADHD, less disruptive, more compensated, or less prototypical presentations may delay support even when developmental need is substantial. More sensitive assessment will require broader behavioral examples, explicit attention to compensation and camouflaging, and multi-informant approaches that capture both external disruption and internal effort.

## Figures and Tables

**Table 1:** From mean-difference thinking to developmental-visibility thinking.

Dimension	Mean-Difference Thinking	Developmental-Visibility Thinking
Core question	Do boys and girls differ, and which group performs better?	When, how and under what social or clinical conditions do sex-related differences become visible?
Primary unit of interpretation	Static group comparison at a task, scale or diagnostic endpoint.	Developmental process linking timing, phenotype and recognition.
View of average effects	Mean differences are treated as evidence of ability difference.	Small average effects are interpreted within highly overlapping distributions and developmentally specific windows.
View of development	Age is often treated as a sufficient developmental marker.	Chronological age, pubertal status, maturation tempo and individual trajectories are modeled together.
Interpretation of phenotype	Observed behavior is often read as a direct index of ability.	Behavior is treated as a context-sensitive output shaped by task demands, strategy use, response style and measurement context.
Role of gendered experience	Often backgrounded or collapsed into the grouping variable.	Treated as a cross-cutting process that may alter self-concept, task engagement, evaluative interaction, stress response and symptom reporting.
Interpretation of clinical ratios	Observed diagnosis ratios are sometimes treated as proxies for underlying liability.	Clinical recognition is treated as an endpoint shaped by informants, referral thresholds, compensatory behavior and diagnostic tool design.
Main methodological risk	Collapsing developmental timing, phenotype and clinical recognition into one binary contrast.	Overextending beyond the measured layer; the framework requires explicit layer-specific interpretation.
Implication for this review	A catalogue of sex-related differences across domains.	An integrative account of how developmental differences become expressed as phenotype and recognized in clinical systems.

Note. This table contrasts two interpretive approaches and is intended as a conceptual summary rather than an exhaustive classification.

**Table 2:** Strength of evidence across the developmental-visibility framework.

Framework Component	Evidence Strength	Main Evidence Base	Interpretation in This Review
Developmental timing	Relatively strong	Longitudinal MRI, diffusion imaging, puberty-sensitive studies, neurophysiology, large developmental cohorts	Sex-related differences are most defensibly interpreted as timing, tempo, pubertal coupling, and variability rather than fixed ability differences.
Phenotypic expression	Moderate and domain-dependent	Reviews, meta-analyses, behavioral tasks, selected developmental studies	Behavioral findings are interpreted as task- and context-sensitive outputs rather than direct readouts of stable sex differences.
Gendered social shaping	Conceptually strong but empirically less integrated	Social-developmental, educational, and experimental evidence on stereotypes, self-concept, motivation, and evaluation	Gendered experience is treated as a measurable mechanism that may shape engagement, strategy, response style, stress, and symptom reporting, but it requires more direct longitudinal testing.
Clinical visibility in ASD/ADHD	Moderate to strong within ASD/ADHD	Studies of camouflaging, compensation, informant discrepancy, referral filtering, diagnostic delay, and tool sensitivity	Recognition is interpreted as a system-level endpoint shaped by observers, tools, and thresholds, not as a transparent indicator of underlying burden.
Full timing–phenotype–recognition sequence	Emerging/hypothesis-generating	Indirect integration across the literature; few studies test all layers together	The framework provides a testable research agenda rather than an already established causal model.

**Table 3:** Layer-by-layer structure of the developmental-visibility framework.

Framework Component	What It Captures	Indicative Evidence Base	Common Interpretive Risk	Methodological Implication
Layer 1: Developmental timing	Age-related trajectories, pubertal coupling, maturation tempo, sensitive windows and interindividual variability.	Longitudinal MRI; diffusion imaging; puberty-sensitive studies; neurophysiology; large developmental cohorts.	Interpreting shifted timing as global superiority, earlier maturity or fixed neuroanatomical category.	Model age and pubertal status together; estimate nonlinear trajectories and variability, not only mean differences.
Layer 2: Phenotypic expression	Observable behavior across language, executive function, emotion and social cognition; task demands, strategy use and response style.	Systematic reviews and meta-analyses; selected longitudinal, behavioral and task-based neuroimaging studies.	Treating a task score as a stable ability difference or assuming that similar performance reflects the same process.	Use process-sensitive measures, including error patterns, strategy reports, reaction-time dynamics, repeated assessments and neural or physiological indicators.
Cross-cutting process: Gendered social shaping	Self-concept, task engagement, evaluative interaction, stereotype salience, stress responding and symptom reporting.	Social cognitive theory; developmental intergroup theory; studies of gendered self-concept, stereotype salience and domain engagement.	Treating social context as a background variable rather than as part of how phenotype is recognized.	Measure gendered experience directly through self-concept, parent/teacher beliefs, peer norms, classroom expectations and broader cultural context.
Layer 3: Clinical visibility/recognition	Informant perception, parent/teacher report, referral thresholds, diagnostic tool design, compensation, camouflaging and delayed recognition.	ASD and ADHD studies on female phenotype, camouflaging, referral filtering, informant discrepancy and diagnostic delay.	Reading observed diagnosis ratios as transparent indicators of underlying prevalence or severity.	Compare community and clinic samples; use multi-informant designs; test tool sensitivity to compensated or internally effortful presentations.
Integrative research agenda	How developmental timing, behavioral process, social experience, and recognition interact across development.	Combined evidence across developmental neuroscience, cognitive development, gendered experience, and clinical recognition research.	Studying one layer in isolation and generalizing beyond it.	Use longitudinal, multi-level designs that separate sex, gender, and gendered experience and test measurement invariance across groups and informants.

**Table 4:** Linking gendered social mechanisms to measurable neurocognitive and behavioral outcomes.

Social Mechanism	Proximal Process	Measurable Indicators	Relevant Outcome Domains
Stereotype salience	Task threat, identity congruence, evaluative concern	Response latency, error monitoring, persistence, performance variability, self-reported task anxiety	Executive function, academic cognition, emotion regulation
Gendered self-concept	Confidence, perceived competence, domain belonging	Self-concept scales, willingness to attempt difficult items, strategy reports, task engagement	Language, mathematics-related cognition, social cognition
Evaluative interaction	Behavior under observation by adults or peers	Cautious responding, disengagement, overcompensation, observer-rated regulation	Executive function, emotion expression, social behavior
Adult expectations	Interpretation of behavior and referral concern	Parent/teacher discrepancy, referral likelihood, symptom ratings, diagnostic concern	ADHD recognition, ASD recognition, internalizing/externalizing presentations
Compensation and camouflaging reinforcement	Outward performance despite internal effort	Self-report–observer mismatch, fatigue, internal distress, delayed referral	ASD/ADHD clinical visibility, social cognition

**Table 5:** Practical applications of the developmental-visibility framework.

Application Area	Practical Question	Example Implementation
Clinical assessment	Is the child’s difficulty externally visible, internally effortful, or context-dependent?	Ask about compensation, fatigue, camouflaging, school–home differences, and internal distress.
Multi-informant evaluation	Do parents, teachers, and children report different parts of the phenotype?	Treat informant discrepancy as clinically meaningful rather than as simple error.
ASD/ADHD recognition	Is lower disruption masking developmental burden?	Assess inattentive, socially compensated, or internally effortful presentations.
Developmental research	Is the observed difference a static group difference or a timing effect?	Model age, pubertal status, nonlinear trajectories, and within-group variability.
Cognitive research	Does similar performance reflect similar processes?	Include reaction time, error patterns, strategy reports, and task engagement measures.
Clinical-recognition research	Who enters assessment systems and why?	Compare community, school, and clinic samples; test measurement invariance and referral pathways.

## Data Availability

No new data were created or analyzed in this study.

## Supplementary Materials

The following supporting information can be downloaded at: https://www.mdpi.com/article/10.3390/children13060725/s1, Figure S1: Flow diagram of study identification, screening, and inclusion; Table S1: Search strategy; Table S2: Final scope and summary; Table S3: Characteristics of included primary empirical studies (n = 76); Table S4: Characteristics of included review and synthesis sources (n = 34). The supplementary file includes the full search strings, search dates, screening flow, and evidence charting table.

## Abbreviations

ADHD	Attention-deficit/hyperactivity disorder
ASD	Autism spectrum disorder
DTI	Diffusion tensor imaging
EF	Executive function
MRI	Magnetic resonance imaging
STEM	Science, technology, engineering, and mathematics

## References

[1] Gilmore J.H., Knickmeyer R.C., Gao W. (2018). Imaging Structural and Functional Brain Development in Early Childhood. Nat. Rev. Neurosci..

[2] Dong H.-M., Margulies D.S., Zuo X.-N., Holmes A.J. (2021). Shifting Gradients of Macroscale Cortical Organization Mark the Transition from Childhood to Adolescence. Proc. Natl. Acad. Sci. USA.

[3] Kaczkurkin A.N., Raznahan A., Satterthwaite T.D. (2019). Sex Differences in the Developing Brain: Insights from Multimodal Neuroimaging. Neuropsychopharmacology.

[4] Lenroot R.K., Giedd J.N. (2010). Sex Differences in the Adolescent Brain. Brain Cogn..

[5] Lenroot R.K., Gogtay N., Greenstein D.K., Wells E.M., Wallace G.L., Clasen L.S., Blumenthal J.D., Lerch J., Zijdenbos A.P., Evans A.C. (2007). Sexual Dimorphism of Brain Developmental Trajectories During Childhood and Adolescence. NeuroImage.

[6] Beck D., Ferschmann L., MacSweeney N., Norbom L.B., Wiker T., Aksnes E., Karl V., Dégeilh F., Holm M., Mills K.L. (2023). Puberty Differentially Predicts Brain Maturation in Male and Female Youth: A Longitudinal ABCD Study. Dev. Cogn. Neurosci..

[7] Giedd J.N., Raznahan A., Mills K.L., Lenroot R.K. (2012). Magnetic Resonance Imaging of Male/Female Differences in Human Adolescent Brain Anatomy. Biol. Sex Differ..

[8] Eliot L., Ahmed A., Khan H., Patel J. (2021). Dump the “Dimorphism”: Comprehensive Synthesis of Human Brain Studies Reveals Few Male-Female Differences Beyond Size. Neurosci. Biobehav. Rev..

[9] Genon S., Eickhoff S.B., Kharabian S. (2022). Linking Interindividual Variability in Brain Structure to Behaviour. Nat. Rev. Neurosci..

[10] Marek S., Tervo-Clemmens B., Calabro F.J., Montez D.F., Kay B.P., Hatoum A.S., Donohue M.R., Foran W., Miller R.L., Hendrickson T.J. (2022). Reproducible Brain-Wide Association Studies Require Thousands of Individuals. Nature.

[11] Hyde J.S. (2005). The Gender Similarities Hypothesis. Am. Psychol..

[12] Joel D., Berman Z., Tavor I., Wexler N., Gaber O., Stein Y., Shefi N., Pool J., Urchs S., Margulies D.S. (2015). Sex Beyond the Genitalia: The Human Brain Mosaic. Proc. Natl. Acad. Sci. USA.

[13] Wierenga L.M., Sexton J.A., Laake P., Giedd J.N., Tamnes C.K. (2018). The Pediatric Imaging, Neurocognition, and Genetics Study. A Key Characteristic of Sex Differences in the Developing Brain: Greater Variability in Brain Structure of Boys Than Girls. Cereb. Cortex.

[14] Zell E., Krizan Z., Teeter S.R. (2015). Evaluating Gender Similarities and Differences Using Metasynthesis. Am. Psychol..

[15] Bottenhorn K.L., Cardenas-Iniguez C., Mills K.L., Laird A.R., Herting M.M. (2023). Profiling Intra-and Inter-Individual Differences in Brain Development across Early Adolescence. NeuroImage.

[16] Mills K.L., Siegmund K.D., Tamnes C.K., Ferschmann L., Wierenga L.M., Bos M.G., Luna B., Li C., Herting M.M. (2021). Inter-Individual Variability in Structural Brain Development from Late Childhood to Young Adulthood. NeuroImage.

[17] Herting M.M., Sowell E.R. (2017). Puberty and Structural Brain Development in Humans. Front. Neuroendocrinol..

[18] Ladouceur C.D., Peper J.S., Crone E.A., Dahl R.E. (2012). White Matter Development in Adolescence: The Influence of Puberty and Implications for Affective Disorders. Dev. Cogn. Neurosci..

[19] Piekarski D.J., Colich N.L., Ho T.C. (2023). The Effects of Puberty and Sex on Adolescent White Matter Development: A Systematic Review. Dev. Cogn. Neurosci..

[20] Chaplin T.M., Aldao A. (2013). Gender Differences in Emotion Expression in Children: A Meta-Analytic Review. Psychol. Bull..

[21] Etchell A., Adhikari A., Weinberg L.S., Choo A.L., Garnett E.O., Chow H.M., Chang S.-E. (2018). A Systematic Literature Review of Sex Differences in Childhood Language and Brain Development. Neuropsychologia.

[22] Thompson A.E., Voyer D. (2014). Sex Differences in the Ability to Recognise Non-Verbal Displays of Emotion: A Meta-Analysis. Cogn. Emot..

[23] Cook J., Hull L., Crane L., Mandy W. (2021). Camouflaging in Autism: A Systematic Review. Clin. Psychol. Rev..

[24] Rucklidge J.J. (2010). Gender Differences in Attention-Deficit/Hyperactivity Disorder. Psychiatr. Clin..

[25] Bigler R.S., Liben L.S. (2007). Developmental Intergroup Theory: Explaining and Reducing Children’s Social Stereotyping and Prejudice. Curr. Dir. Psychol. Sci..

[26] Rippon G., Jordan-Young R., Kaiser A., Fine C. (2014). Recommendations for Sex/Gender Neuroimaging Research: Key Principles and Implications for Research Design, Analysis, and Interpretation. Front. Hum. Neurosci..

[27] Kreiser N.L., White S.W. (2014). ASD in Females: Are We Overstating the Gender Difference in Diagnosis?. Clin. Child Fam. Psychol. Rev..

[28] Loyer Carbonneau M., Demers M., Bigras M., Guay M.-C. (2021). Meta-Analysis of Sex Differences in Adhd Symptoms and Associated Cognitive Deficits. J. Atten. Disord..

[29] Zahn-Waxler C., Shirtcliff E.A., Marceau K. (2008). Disorders of Childhood and Adolescence: Gender and Psychopathology. Annu. Rev. Clin. Psychol..

[30] Rutter M., Caspi A., Fergusson D., Horwood L.J., Goodman R., Maughan B., Moffitt T.E., Meltzer H., Carroll J. (2004). Sex Differences in Developmental Reading Disability: New Findings from 4 Epidemiological Studies. JAMA.

[31] Henschel S., Jansen M., Schneider R. (2023). How Gender Stereotypes of Students and Significant Others Are Related to Motivational and Affective Outcomes in Mathematics at the End of Secondary School. Contemp. Educ. Psychol..

[32] Master A., Meltzoff A.N., Tang D., Cheryan S. (2025). Divergence in Children’s Gender Stereotypes and Motivation across Stem Fields. Proc. Natl. Acad. Sci. USA.

[33] Miller D.I., Lauer J.E., Tanenbaum C., Burr L. (2024). The Development of Children’s Gender Stereotypes About Stem and Verbal Abilities: A Preregistered Meta-Analytic Review of 98 Studies. Psychol. Bull..

[34] Bian L., Leslie S.-J., Cimpian A. (2017). Gender Stereotypes About Intellectual Ability Emerge Early and Influence Children’s Interests. Science.

[35] Cvencek D., Meltzoff A.N., Greenwald A.G. (2011). Math–Gender Stereotypes in Elementary School Children. Child Dev..

[36] Del Río M.F., Strasser K., Cvencek D., Susperreguy M.I., Meltzoff A.N. (2019). Chilean Kindergarten Children’s Beliefs About Mathematics: Family Matters. Dev. Psychol..

[37] Wang M.-T., Degol J.L. (2017). Gender Gap in Science, Technology, Engineering, and Mathematics (Stem): Current Knowledge, Implications for Practice, Policy, and Future Directions. Educ. Psychol. Rev..

[38] Watt H.M. (2004). Development of Adolescents’ Self-Perceptions, Values, and Task Perceptions According to Gender and Domain in 7th-through 11th-Grade Australian Students. Child Dev..

[39] Tomasetto C., Alparone F.R., Cadinu M. (2011). Girls’ Math Performance under Stereotype Threat: The Moderating Role of Mothers’ Gender Stereotypes. Dev. Psychol..

[40] Hilliard L.J., Liben L.S. (2010). Differing Levels of Gender Salience in Preschool Classrooms: Effects on Children’s Gender Attitudes and Intergroup Bias. Child Dev..

[41] Bhargava A., Arnold A.P., Bangasser D.A., Denton K.M., Gupta A., Hilliard Krause L.M., Mayer E.A., McCarthy M., Miller W.L., Raznahan A. (2021). Considering Sex as a Biological Variable in Basic and Clinical Studies: An Endocrine Society Scientific Statement. Endocr. Rev..

[42] Clayton J.A., Tannenbaum C. (2016). Reporting Sex, Gender, or Both in Clinical Research?. JAMA.

[43] Heidari S., Babor T.F., De Castro P., Tort S., Curno M. (2016). Sex and Gender Equity in Research: Rationale for the Sager Guidelines and Recommended Use. Res. Integr. Peer Rev..

[44] Mills K.L., Goddings A.-L., Herting M.M., Meuwese R., Blakemore S.-J., Crone E.A., Dahl R.E., Güroğlu B., Raznahan A., Sowell E.R. (2016). Structural Brain Development between Childhood and Adulthood: Convergence across Four Longitudinal Samples. NeuroImage.

[45] De Bellis M.D., Keshavan M.S., Beers S.R., Hall J., Frustaci K., Masalehdan A., Noll J., Boring A.M. (2001). Sex Differences in Brain Maturation During Childhood and Adolescence. Cereb. Cortex.

[46] Forde N.J., Ronan L., Zwiers M.P., Schweren L.J., Alexander-Bloch A.F., Franke B., Faraone S.V., Oosterlaan J., Heslenfeld D.J., Hartman C.A. (2017). Healthy Cortical Development through Adolescence and Early Adulthood. Brain Struct. Funct..

[47] Ingalhalikar M., Smith A., Parker D., Satterthwaite T.D., Elliott M.A., Ruparel K., Hakonarson H., Gur R.E., Gur R.C., Verma R. (2014). Sex Differences in the Structural Connectome of the Human Brain. Proc. Natl. Acad. Sci. USA.

[48] Mutlu A.K., Schneider M., Debbané M., Badoud D., Eliez S., Schaer M. (2013). Sex Differences in Thickness, and Folding Developments Throughout the Cortex. NeuroImage.

[49] Schmithorst V.J., Holland S.K., Dardzinski B.J. (2008). Developmental Differences in White Matter Architecture between Boys and Girls. Hum. Brain Mapp..

[50] Shanmugan S., Seidlitz J., Cui Z., Adebimpe A., Bassett D.S., Bertolero M.A., Davatzikos C., Fair D.A., Gur R.E., Gur R.C. (2022). Sex Differences in the Functional Topography of Association Networks in Youth. Proc. Natl. Acad. Sci. USA.

[51] Herting M.M., Gautam P., Spielberg J.M., Dahl R.E., Sowell E.R. (2015). A Longitudinal Study: Changes in Cortical Thickness and Surface Area During Pubertal Maturation. PLoS ONE.

[52] Herting M.M., Maxwell E.C., Irvine C., Nagel B.J. (2012). The Impact of Sex, Puberty, and Hormones on White Matter Microstructure in Adolescents. Cereb. Cortex.

[53] Neufang S., Specht K., Hausmann M., Güntürkün O., Herpertz-Dahlmann B., Fink G.R., Konrad K. (2009). Sex Differences and the Impact of Steroid Hormones on the Developing Human Brain. Cereb. Cortex.

[54] Peper J.S., Brouwer R.M., Schnack H.G., van Baal G.C., van Leeuwen M., van den Berg S.M., Delemarre-Van de Waal H.A., Boomsma D.I., Kahn R.S., Pol H.E.H. (2009). Sex Steroids and Brain Structure in Pubertal Boys and Girls. Psychoneuroendocrinology.

[55] Satterthwaite T.D., Vandekar S., Wolf D.H., Ruparel K., Roalf D.R., Jackson C., Elliott M.A., Bilker W.B., Calkins M.E., Prabhakaran K. (2014). Sex Differences in the Effect of Puberty on Hippocampal Morphology. J. Am. Acad. Child Adolesc. Psychiatry.

[56] Van Duijvenvoorde A.C., Westhoff B., de Vos F., Wierenga L.M., Crone E.A. (2019). A Three-Wave Longitudinal Study of Subcortical–Cortical Resting-State Connectivity in Adolescence: Testing Age-and Puberty-Related Changes. Hum. Brain Mapp..

[57] Wiglesworth A., Fiecas M.B., Xu M., Neher A.T., Padilla L., Carosella K.A., Roediger D.J., Mueller B.A., Luciana M., Klimes-Dougan B. (2023). Sex and Age Variations in the Impact of Puberty on Cortical Thickness and Associations with Internalizing Symptoms and Suicidal Ideation in Early Adolescence. Dev. Cogn. Neurosci..

[58] Bava S., Boucquey V., Goldenberg D., Thayer R.E., Ward M., Jacobus J., Tapert S.F. (2011). Sex Differences in Adolescent White Matter Architecture. Brain Res..

[59] Geeraert B.L., Lebel R.M., Lebel C. (2019). A Multiparametric Analysis of White Matter Maturation During Late Childhood and Adolescence. Hum. Brain Mapp..

[60] Pangelinan M.M., Leonard G., Perron M., Pike G.B., Richer L., Veillette S., Pausova Z., Paus T. (2016). Puberty and Testosterone Shape the Corticospinal Tract During Male Adolescence. Brain Struct. Funct..

[61] Seunarine K.K., Clayden J.D., Jentschke S., Muñoz M., Cooper J.M., Chadwick M.J., Banks T., Vargha-Khadem F., Clark C.A. (2016). Sexual Dimorphism in White Matter Developmental Trajectories Using Tract-Based Spatial Statistics. Brain Connect..

[62] Barendse M.E.A., Swartz J.R., Taylor S.L., Fine J.R., Shirtcliff E.A., Yoon L., McMillan S.J., Tully L.M., Guyer A.E. (2024). Sex and Pubertal Variation in Reward-Related Behavior and Neural Activation in Early Adolescents. Dev. Cogn. Neurosci..

[63] Killgore W.D., Oki M., Yurgelun-Todd D.A. (2001). Sex-Specific Developmental Changes in Amygdala Responses to Affective Faces. Neuroreport.

[64] Ott L.R., Penhale S.H., Taylor B.K., Lew B.J., Wang Y.-P., Calhoun V.D., Stephen J.M., Wilson T.W. (2021). Spontaneous Cortical Meg Activity Undergoes Unique Age-and Sex-Related Changes During the Transition to Adolescence. NeuroImage.

[65] Picci G., Ott L.R., Penhale S.H., Taylor B.K., Johnson H.J., Willett M.P., Okelberry H.J., Wang Y.P., Calhoun V.D., Stephen J.M. (2023). Developmental Changes in Endogenous Testosterone Have Sexually-Dimorphic Effects on Spontaneous Cortical Dynamics. Hum. Brain Mapp..

[66] Schmithorst V.J., Holland S.K. (2006). Functional Mri Evidence for Disparate Developmental Processes Underlying Intelligence in Boys and Girls. NeuroImage.

[67] Schmithorst V.J., Holland S.K. (2007). Sex Differences in the Development of Neuroanatomical Functional Connectivity Underlying Intelligence Found Using Bayesian Connectivity Analysis. NeuroImage.

[68] Corbett B.A., Vandekar S., Muscatello R.A., Tanguturi Y. (2020). Pubertal Timing During Early Adolescence: Advanced Pubertal Onset in Females with Autism Spectrum Disorder. Autism Res..

[69] Groenman A.P., van der Oord S., Geurts H.M. (2024). Navigating Adolescence: Pubertal Development in Autism Spectrum Conditions and Its Relation to Mental Health. Arch. Women’s Ment. Health.

[70] Tsompanidis A., Hampton S., Aydin E., Allison C., Holt R., Baron-Cohen S. (2023). Mini-Puberty Testosterone and Infant Autistic Traits. Front. Endocrinol..

[71] Adani S., Cepanec M. (2019). Sex Differences in Early Communication Development: Behavioral and Neurobiological Indicators of More Vulnerable Communication System Development in Boys. Croat. Med. J..

[72] Gaillard A., Fehring D.J., Rossell S.L. (2021). Sex Differences in Executive Control: A Systematic Review of Functional Neuroimaging Studies. Eur. J. Neurosci..

[73] Taddei M., Bulgheroni S., Riva D., Erbetta A. (2023). Task-Related Functional Neuroimaging Contribution to Sex/Gender Differences in Cognition and Emotion During Development. J. Neurosci. Res..

[74] Leaper C., Smith T.E. (2004). A Meta-Analytic Review of Gender Variations in Children’s Language Use: Talkativeness, Affiliative Speech, and Assertive Speech. Dev. Psychol..

[75] Boelema S.R., Harakeh Z., Ormel J., Hartman C.A., Vollebergh W.A., Van Zandvoort M.J. (2014). Executive Functioning Shows Differential Maturation from Early to Late Adolescence: Longitudinal Findings from a TRAILS Study. Neuropsychology.

[76] Roalf D.R., Gur R.E., Ruparel K., Calkins M.E., Satterthwaite T.D., Bilker W.B., Hakonarson H., Harris L.J., Gur R.C. (2014). Within-Individual Variability in Neurocognitive Performance: Age-and Sex-Related Differences in Children and Youths from Ages 8 to 21. Neuropsychology.

[77] Doidge J.L., Flora D.B., Toplak M.E. (2021). A Meta-Analytic Review of Sex Differences on Delay of Gratification and Temporal Discounting Tasks in Adhd and Typically Developing Samples. J. Atten. Disord..

[78] Silverman I.W. (2021). Gender Differences in Inhibitory Control as Assessed on Simple Delay Tasks in Early Childhood: A Meta-Analysis. Int. J. Behav. Dev..

[79] Del Piero L.B., Saxbe D.E., Margolin G. (2016). Basic Emotion Processing and the Adolescent Brain: Task Demands, Analytic Approaches, and Trajectories of Changes. Dev. Cogn. Neurosci..

[80] Ordaz S., Luna B. (2012). Sex Differences in Physiological Reactivity to Acute Psychosocial Stress in Adolescence. Psychoneuroendocrinology.

[81] Ullsperger J.M., Nikolas M.A. (2017). A Meta-Analytic Review of the Association between Pubertal Timing and Psychopathology in Adolescence: Are There Sex Differences in Risk?. Psychol. Bull..

[82] Mestre M.V., Samper P., Frías M.D., Tur A.M. (2009). Are Women More Empathetic Than Men? A Longitudinal Study in Adolescence. Span. J. Psychol..

[83] Van der Graaff J., Branje S., De Wied M., Hawk S., Van Lier P., Meeus W. (2014). Perspective Taking and Empathic Concern in Adolescence: Gender Differences in Developmental Changes. Dev. Psychol..

[84] Van der Graaff J., Carlo G., Crocetti E., Koot H.M., Branje S. (2018). Prosocial Behavior in Adolescence: Gender Differences in Development and Links with Empathy. J. Youth Adolesc..

[85] Xiao S.X., Hashi E.C., Korous K.M., Eisenberg N. (2019). Gender Differences across Multiple Types of Prosocial Behavior in Adolescence: A Meta-Analysis of the Prosocial Tendency Measure-Revised (Ptm-R). J. Adolesc..

[86] Hull L., Mandy W., Petrides K. (2017). Behavioural and Cognitive Sex/Gender Differences in Autism Spectrum Condition and Typically Developing Males and Females. Autism.

[87] Parish-Morris J., Liberman M.Y., Cieri C., Herrington J.D., Yerys B.E., Bateman L., Donaher J., Ferguson E., Pandey J., Schultz R.T. (2017). Linguistic Camouflage in Girls with Autism Spectrum Disorder. Mol. Autism.

[88] Tubío-Fungueiriño M., Cruz S., Sampaio A., Carracedo A., Fernández-Prieto M. (2021). Social Camouflaging in Females with Autism Spectrum Disorder: A Systematic Review. J. Autism Dev. Disord..

[89] Wood-Downie H., Wong B., Kovshoff H., Cortese S., Hadwin J.A. (2021). Research Review: A Systematic Review and Meta-Analysis of Sex/Gender Differences in Social Interaction and Communication in Autistic and Nonautistic Children and Adolescents. J. Child Psychol. Psychiatry.

[90] Brumariu L.E., Nair T.K., Waslin S.M., Rodrigues G.A., Moore M.T., Kerns K.A. (2025). Parental Emotion Socialization and Internalizing Problems in Childhood and Adolescence: A Meta-Analytic Review. Dev. Psychopathol..

[91] Song A., Cola M., Plate S., Petrulla V., Yankowitz L., Pandey J., Schultz R.T., Parish-Morris J. (2021). Natural Language Markers of Social Phenotype in Girls with Autism. J. Child Psychol. Psychiatry.

[92] Ratto A.B., Kenworthy L., Yerys B.E., Bascom J., Wieckowski A.T., White S.W., Wallace G.L., Pugliese C., Schultz R.T., Ollendick T.H. (2018). What About the Girls? Sex-Based Differences in Autistic Traits and Adaptive Skills. J. Autism Dev. Disord..

[93] Rynkiewicz A., Schuller B., Marchi E., Piana S., Camurri A., Lassalle A., Baron-Cohen S. (2016). An Investigation of the ‘Female Camouflage Effect’ in Autism Using a Computerized Ados-2 and a Test of Sex/Gender Differences. Mol. Autism.

[94] Ercan E.S., Tahıllıoğlu A., Tufan A.E., Bilaç Ö. (2025). Teachers Predict Adhd More Accurately Than Parents: Findings from a Large Epidemiological Survey. Nord. J. Psychiatry.

[95] Sciutto M.J., Nolfi C.J., Bluhm C. (2004). Effects of Child Gender and Symptom Type on Referrals for Adhd by Elementary School Teachers. J. Emot. Behav. Disord..

[96] Madsen K.B., Ravn M.H., Arnfred J., Olsen J., Rask C.U., Obel C. (2018). Characteristics of Undiagnosed Children with Parent-Reported Adhd Behaviour. Eur. Child Adolesc. Psychiatry.

[97] Mowlem F.D., Rosenqvist M.A., Martin J., Lichtenstein P., Asherson P., Larsson H. (2019). Sex Differences in Predicting Adhd Clinical Diagnosis and Pharmacological Treatment. Eur. Child Adolesc. Psychiatry.

[98] Ramtekkar U.P., Reiersen A.M., Todorov A.A., Todd R.D. (2010). Sex and Age Differences in Attention-Deficit/Hyperactivity Disorder Symptoms and Diagnoses: Implications for Dsm-V and Icd-11. J. Am. Acad. Child Adolesc. Psychiatry.

[99] Diener T.-S.L., Jackson M., Lee M.A., Grové C., Nguyen V. (2025). Cross-Cultural Disparities in Teachers’ Reports of Adhd Symptoms and Behavior: A Scoping Review. Soc. Psychol. Educ..

[100] Gross I.M., Gao Y., Lee M.J., Hipwell A.E., Keenan K. (2024). The Adhd Phenotype in Black and White Girls from Childhood to Adolescence: Results from the Community-Based Pittsburgh Girls Study. J. Atten. Disord..

[101] Miller D.I., Nolla K.M., Eagly A.H., Uttal D.H. (2018). The Development of Children’s Gender-Science Stereotypes: A Meta-Analysis of 5 Decades of Us Draw-a-Scientist Studies. Child Dev..

[102] De Los Reyes A., Augenstein T.M., Wang M., Thomas S.A., Drabick D.A., Burgers D.E., Rabinowitz J. (2015). The Validity of the Multi-Informant Approach to Assessing Child and Adolescent Mental Health. Psychol. Bull..

