# Supplementary material for: From Developmental Timing to Clinical Visibility: An Integrative Narrative Review of Sex-Related Neurocognitive Development
Source: Children (Basel). 2026 May 23;13(6):725. doi: 10.3390/children13060725 (PMC13297654; doi:10.3390/children13060725)
Supplement: Supplementary file 1 [file children-13-00725-s001.zip › children-4331004-supplementary.pdf]

Sex-related differences in neurocognitive development in children and adolescents: developmental timing, phenotypic expression, and clinical visibility

Supplementary Table S1. Search strategy

PubMed Flow A：Developmental timing

Step	Search Terms	Outcome
#1	("sex differences"[Title/Abstract] OR "sex-related differences"[Title/Abstract] OR "gender differences"[Title/Abstract])	92074
#2	("Child"[Mesh] OR "Adolescent"[Mesh] OR "Puberty"[Mesh] OR child*[Title/Abstract] OR adolescen*[Title/Abstract] OR youth[Title/Abstract] OR pubert*[Title/Abstract] OR Tanner[Title/Abstract])	3,668,882
#3	("brain development"[Title/Abstract] OR "brain maturation"[Title/Abstract] OR neurodevelopment*[Title/Abstract] OR "developmental timing"[Title/Abstract] OR "pubertal timing"[Title/Abstract] OR trajectory[Title/Abstract] OR trajectories[Title/Abstract] OR variability[Title/Abstract] OR "cortical thickness"[Title/Abstract] OR "surface area"[Title/Abstract] OR "white matter"[Title/Abstract] OR neuroimaging[Title/Abstract] OR MRI[Title/Abstract] OR fMRI[Title/Abstract] OR DTI[Title/Abstract] OR longitudinal[Title/Abstract])	1,602,382
#4	NOT (animals[mh] NOT humans[mh])	4,981,089
#5	(#1 AND #2 AND #3 AND #4)	4709

PubMed Flow B：Phenotypic expression

Step	Search Terms	Outcome
#1	("sex differences"[Title/Abstract] OR "sex-related differences"[Title/Abstract] OR "gender differences"[Title/Abstract])	92074
#2	("Child"[Mesh] OR "Adolescent"[Mesh] OR child*[Title/Abstract] OR adolescen*[Title/Abstract] OR youth[Title/Abstract])	3,649,725
#3	(("language development"[Title/Abstract] OR language[Title/Abstract] OR communication[Title/Abstract]) OR ("executive function*"[Title/Abstract] OR "cognitive control"[Title/Abstract]) OR ("emotion recognition"[Title/Abstract] OR "emotion expression"[Title/Abstract] OR emotion*[Title/Abstract]) OR ("social cognition"[Title/Abstract] OR empathy[Title/Abstract] OR prosocial[Title/Abstract]))	976,026
#4	(#1 AND #2 AND #3)	3651

PubMed Flow C：Clinical visibility in ASD/ADHD

Step	Search Terms	Outcome
#1	(autis*[Title/Abstract] OR ASD[Title/Abstract] OR ADHD[Title/Abstract] OR "attention deficit hyperactivity disorder"[Title/Abstract])	133,024
#2	("sex differences"[Title/Abstract] OR "sex-related differences"[Title/Abstract] OR "gender differences"[Title/Abstract] OR female[Title/Abstract] OR girls[Title/Abstract])	92074
#3	(camouflaging[Title/Abstract] OR compensation[Title/Abstract] OR "recognition bias"[Title/Abstract] OR "referral bias"[Title/Abstract] OR "diagnostic bias"[Title/Abstract] OR "clinical visibility"[Title/Abstract] OR underdiagnos*[Title/Abstract] OR underidentif*[Title/Abstract] OR inattenti*[Title/Abstract] OR hyperactiv*[Title/Abstract]	170,527

	)	
#4	("Child"[Mesh] OR "Adolescent"[Mesh] OR child*[Title/Abstract] OR adolescen*[Title/Abstract] OR pediatric*[Title/Abstract] OR youth[Title/Abstract]))	3,733,405
#5	(#1 AND #2 AND #3 AND #4)	529

WoS Flow A：Developmental timing (n = 10568)

TS=(("sex differences" OR "sex-related differences" OR "gender differences")

AND

(child* OR adolescen* OR youth OR pubert* OR Tanner)

AND

("brain development" OR "brain maturation" OR neurodevelopment*

OR "developmental timing" OR "pubertal timing"

OR trajectory OR trajectories OR variability

OR "cortical thickness" OR "surface area" OR "white matter"

OR neuroimaging OR MRI OR fMRI OR DTI

OR longitudinal

)

)

Flow B：Phenotypic expression (n = 802)

```
TS=(
  ("sex differences" OR "sex-related differences" OR "gender differences")
  AND
  (child* OR adolescen* OR youth)
  AND
  (
    ("language development" OR language OR communication)
    OR
    ("executive function*" OR "cognitive control")
    OR
    ("emotion recognition" OR "emotion expression" OR emotion*)
    OR
    ("social cognition" OR empathy OR prosocial)
  )
  AND
  ("systematic review" OR meta-analy* OR review)
)
```

Flow C：Clinical visibility in ASD/ADHD (n = 5495)

```
TS=(
  (autis* OR ASD OR ADHD OR "attention deficit hyperactivity disorder")
  AND
  ("sex differences" OR "sex-related differences" OR "gender differences" OR female OR girls)
  AND
  (
    camouflaging OR compensation
    OR "recognition bias" OR "referral bias"
    OR "diagnostic bias" OR "clinical visibility"
    OR underdiagnos* OR underidentif*
    OR inattenti* OR hyperactiv*
  )
  AND
  (child* OR adolescen* OR pediatric* OR youth)
)
```

Supplementary Table S2A. Final scope and summary

Flow	Primary empirical (PubMed)	Primary empirical (WoS unique)	Reviews / syntheses (PubMed + WoS unique)	Total unique included
Flow A: Developmental timing	36	12	9	57
Flow B: Phenotypic expression	3	0	14	17
Flow C: Clinical visibility in ASD/ADHD	15	10	11	36
Total	54	22	34	110

Supplementary Table S2B. Operational eligibility rules

Population. Human participants in childhood, adolescence, or transition-aged youth when the developmental process of interest clearly began in childhood/adolescence. Both general-population and clinical samples were eligible when directly relevant to the review question.

Flow A (Developmental timing). Records had to address sex-related or gender-related differences in developmental timing, pubertal coupling, maturation tempo, developmental trajectories, or inter-individual variability in brain or neurocognitive development.

Flow B (Phenotypic expression). Records had to address sex-related or gender-related differences in language/communication, executive function, emotion/emotion recognition/expression, or social cognition/empathy/prosocial behavior during development.

Flow C (Clinical visibility in ASD/ADHD). Records had to address sex-related or gender-related differences in diagnostic visibility, referral filtering, camouflaging, compensation, underrecognition, or clinically meaningful presentation differences in ASD and/or ADHD.

Review-type sources. Systematic reviews, meta-analyses, umbrella reviews, and directly relevant key reviews were retained in a separate review/synthesis section rather than mixed with the primary empirical studies.

Key exclusion rules. Adult-only or aging studies without child/adolescent developmental relevance, animal-only studies, purely somatic/metabolic/orthopedic/sports papers without neurodevelopmental or clinical-visibility endpoints, broad mental-health or educational papers lacking a direct neurocognitive/visibility link, and methods-only pipeline papers without substantive relevance were excluded.

The final database searches were conducted on March 25, 2026, and backward citation tracking was completed on April 10, 2026.

Supplementary Table S2C. Deduplicated master list of included studies.

3.1 Flow A: Developmental timing (48 studies)

1. **[PubMed]** Ingalhalikar M, Smith A, Parker D, Satterthwaite TD, Elliott MA, Ruparel K, et al. (2014). Sex differences in the structural connectome of the human brain. DOI: 10.1073/pnas.1316909110. Reason for inclusion: Included because it directly characterizes sex-related differences in large-scale structural brain connectivity during youth development.
2. **[PubMed]** Beck D, Ferschmann L, MacSweeney N, Norbom LB, Wiker T, Aksnes E, et al. (2023). Puberty differentially predicts brain maturation in male and female youth: A longitudinal ABCD Study. DOI: 10.1016/j.dcn.2023.101261. Reason for inclusion: Included because it explicitly models puberty-related brain maturation and compares male and female developmental trajectories.

3. **[PubMed]** Tomasi D, Volkow ND (2023). Measures of Brain Connectivity and Cognition by Sex in US Children. DOI: 10.1001/jamanetworkopen.2023.0157. Reason for inclusion: Included because it links sex-related differences in childhood brain connectivity to cognitive performance in a large developmental cohort.
4. **[PubMed]** Fish AM, Nadig A, Seidlitz J, Reardon PK, Mankiw C, McDermott CL, et al. (2020). Sex-biased trajectories of amygdalo-hippocampal morphology change over human development. DOI: 10.1016/j.neuroimage.2019.116122. Reason for inclusion: Included because it maps sex-biased developmental trajectories in limbic morphology across childhood and adolescence.
5. **[PubMed]** Sanders AFP, Harms MP, Kandala S, Marek S, Somerville LH, Bookheimer SY, et al. (2023). Age-related differences in resting-state functional connectivity from childhood to adolescence. DOI: 10.1093/cercor/bhad011. Reason for inclusion: Included because it reports age-related network development and tests whether sex moderates those trajectories.
6. **[PubMed]** Shanmugan S, Seidlitz J, Cui Z, Adebimpe A, Bassett DS, Bertolero MA, et al. (2022). Sex differences in the functional topography of association networks in youth. DOI: 10.1073/pnas.2110416119. Reason for inclusion: Included because it addresses sex-differential organization of functional association networks in a youth sample.
7. **[PubMed]** Forde NJ, Ronan L, Zwiers MP, Schweren LJS, Alexander-Bloch AF, Franke B, et al. (2017). Healthy cortical development through adolescence and early adulthood. DOI: 10.1007/s00429-017-1424-0. Reason for inclusion: Included because it examines age and sex effects on multiple cortical morphometric indices during adolescence.
8. **[PubMed]** Lynch KM, Shi Y, Toga AW, Clark KA; Pediatric Imaging, Neurocognition and Genetics Study (2019). Hippocampal Shape Maturation in Childhood and Adolescence. DOI: 10.1093/cercor/bhy244. Reason for inclusion: Included because it evaluates age-related hippocampal maturation and sex interactions in a large developmental sample.
9. **[PubMed]** Mills KL, Siegmund KD, Tamnes CK, Ferschmann L, Wierenga LM, Bos MGN, et al. (2021). Inter-individual variability in structural brain development from late childhood to young adulthood. DOI: 10.1016/j.neuroimage.2021.118450. Reason for inclusion: Included because it quantifies developmental variability and sex differences in structural brain change.
10. **[PubMed]** Satterthwaite TD, Vandekar S, Wolf DH, Ruparel K, Roalf DR, Jackson C, et al. (2014). Sex differences in the effect of puberty on hippocampal morphology. DOI: 10.1016/j.jaac.2013.12.002. Reason for inclusion: Included because it directly tests sex-by-puberty effects on hippocampal development.
11. **[PubMed]** Whittle S, Barendse M, Pozzi E, Vijayakumar N, Simmons JG (2020). Pubertal hormones predict sex-specific trajectories of pituitary gland volume during the transition from childhood to adolescence. DOI: 10.1016/j.neuroimage.2019.116256. Reason for inclusion: Included because it links pubertal hormones with sex-specific neuroanatomical trajectories.
12. **[PubMed]** Picci G, Ott LR, Penhale SH, Taylor BK, Johnson HJ, Willett MP, et al. (2023). Developmental changes in endogenous testosterone have sexually-dimorphic effects on spontaneous cortical dynamics. DOI: 10.1002/hbm.26496. Reason for inclusion: Included because it assesses hormone-linked sex-differential changes in cortical function during development.
13. **[PubMed]** Frere PB, Vetter NC, Artiges E, Filippi I, Miranda R, Vulser H, et al. (2020). Sex effects on structural maturation of the limbic system and outcomes on emotional regulation during adolescence. DOI: 10.1016/j.neuroimage.2019.116441. Reason for inclusion: Included because it examines sex-related limbic maturation in adolescence and relates it to emotion regulation.
14. **[PubMed]** Corrigan NM, Rokem A, Kuhl PK (2024). COVID-19 lockdown effects on adolescent brain structure suggest accelerated maturation that is more pronounced in females than in males. DOI: 10.1073/pnas.2403200121. Reason for inclusion: Included because it addresses sex-differential maturation patterns in an adolescent brain-development context.
15. **[PubMed]** Ott LR, Penhale SH, Taylor BK, Lew BJ, Wang YP, Calhoun VD, et al. (2021). Spontaneous cortical MEG activity undergoes unique age- and sex-related changes during the transition to adolescence. DOI: 10.1016/j.neuroimage.2021.118552. Reason for inclusion: Included because it provides age- and sex-sensitive developmental neurophysiology during the transition to adolescence.
16. **[PubMed]** Schmithorst VJ, Holland SK, Dardzinski BJ (2008). Developmental differences in white matter architecture between boys and girls. DOI: 10.1002/hbm.20431. Reason for inclusion: Included because it is a foundational developmental DTI study of sex-related white-matter maturation.

17. **[PubMed]** Bava S, Boucquey V, Goldenberg D, Thayer RE, Ward M, Jacobus J, et al. (2011). Sex differences in adolescent white matter architecture. DOI: 10.1016/j.brainres.2010.12.051. Reason for inclusion: Included because it further characterizes sex-related developmental white-matter architecture in adolescence.
18. **[PubMed]** Seunarine KK, Clayden JD, Jentschke S, Muñoz M, Cooper JM, Chadwick MJ, et al. (2016). Sexual Dimorphism in White Matter Developmental Trajectories Using Tract-Based Spatial Statistics. DOI: 10.1089/brain.2015.0340. Reason for inclusion: Included because it directly models sexually dimorphic white-matter developmental trajectories.
19. **[PubMed]** Mutlu AK, Schneider M, Debbané M, Badoud D, Eliez S, Schaer M (2013). Sex differences in thickness, and folding developments throughout the cortex. DOI: 10.1016/j.neuroimage.2013.05.076. Reason for inclusion: Included because it examines sex-related developmental changes in cortical thickness and gyrification.
20. **[PubMed]** Mills KL, Goddings AL, Herting MM, Meuwese R, Blakemore SJ, Crone EA, et al. (2016). Structural brain development between childhood and adulthood: Convergence across four longitudinal samples. DOI: 10.1016/j.neuroimage.2016.07.044. Reason for inclusion: Included because it synthesizes longitudinal structural-brain development across cohorts and is central to developmental-timing inference.
21. **[PubMed]** Herting MM, Gautam P, Spielberg JM, Dahl RE, Sowell ER (2015). A longitudinal study: changes in cortical thickness and surface area during pubertal maturation. DOI: 10.1371/journal.pone.0119774. Reason for inclusion: Included because it specifically links pubertal maturation to longitudinal cortical development.
22. **[PubMed]** Herting MM, Johnson C, Mills KL, Vijayakumar N, Dennison M, Liu C, et al. (2018). Development of subcortical volumes across adolescence in males and females: A multisample study of longitudinal changes. DOI: 10.1016/j.neuroimage.2018.01.020. Reason for inclusion: Included because it provides multisample longitudinal evidence on sex-related subcortical developmental change.
23. **[PubMed]** Peper JS, Brouwer RM, Schnack HG, van Baal GC, van Leeuwen M, van den Berg SM, et al. (2009). Sex steroids and brain structure in pubertal boys and girls. DOI: 10.1016/j.psyneuen.2008.09.012. Reason for inclusion: Included because it connects pubertal hormones to sex-related brain-structure variation during adolescence.
24. **[PubMed]** Nguyen TV, McCracken J, Ducharme S, Botteron KN, Mahabir M, Johnson W, et al. (2013). Testosterone-related cortical maturation across childhood and adolescence. DOI: 10.1093/cercor/bhs125. Reason for inclusion: Included because it directly models testosterone-associated cortical maturation across development.
25. **[PubMed]** Herting MM, Kim R, Uban KA, Kan E, Binley A, Sowell ER (2017). Longitudinal changes in pubertal maturation and white matter microstructure. DOI: 10.1016/j.psyneuen.2017.03.017. Reason for inclusion: Included because it longitudinally examines puberty-linked white-matter maturation with explicit sex relevance.
26. **[PubMed]** Herting MM, Maxwell EC, Irvine C, Nagel BJ (2012). The impact of sex, puberty, and hormones on white matter microstructure in adolescents. DOI: 10.1093/cercor/bhr246. Reason for inclusion: Included because it jointly evaluates sex, puberty, and hormone effects on adolescent white matter.
27. **[PubMed]** Wierenga LM, Sexton JA, Laake P, Giedd JN, Tamnes CK; Pediatric Imaging, Neurocognition, et al. (2018). A Key Characteristic of Sex Differences in the Developing Brain: Greater Variability in Brain Structure of Boys than Girls. DOI: 10.1093/cercor/bhx154. Reason for inclusion: Included because it directly addresses sex-related variability as a developmental-timing phenomenon.
28. **[PubMed]** Pangelinan MM, Leonard G, Perron M, Pike GB, Richer L, Veillette S, et al. (2016). Puberty and testosterone shape the corticospinal tract during male adolescence. DOI: 10.1007/s00429-014-0956-9. Reason for inclusion: Included because it addresses hormone-related maturation during adolescence within the developmental-timing framework.
29. **[PubMed]** Campbell IG, Grimm KJ, de Bie E, Feinberg I (2012). Sex, puberty, and the timing of sleep EEG measured adolescent brain maturation. DOI: 10.1073/pnas.1120860109. Reason for inclusion: Included because it uses sleep EEG as an index of adolescent brain maturation and examines sex and puberty jointly.
30. **[PubMed]** Lawrence KE, Abaryan Z, Laltoo E, Hernandez LM, Gandal MJ, McCracken JT, et al. (2023). White matter microstructure shows sex differences in late childhood: Evidence from 6797 children. DOI: 10.1002/hbm.26079. Reason for inclusion: Included because it reports large-sample late-childhood white-matter sex differences relevant to developmental timing.

31. **[PubMed]** Yang X, Li A, Li L, Li T, Li P, Liu M (2021). Multimodal Image Analysis of Sexual Dimorphism in Developing Childhood Brain. DOI: 10.1007/s10548-021-00823-7. Reason for inclusion: Included because it uses multimodal developmental neuroimaging to characterize sexual dimorphism in childhood brain development.
32. **[PubMed]** Kurth F, Schijven D, van den Heuvel OA, Hoogman M, van Rooij D, Stein DJ, et al. (2024). Large-scale analysis of structural brain asymmetries during neurodevelopment: Associations with age and sex in 4265 children and adolescents. DOI: 10.1002/hbm.26754. Reason for inclusion: Included because it examines age- and sex-related asymmetry during neurodevelopment in a large youth sample.
33. **[PubMed]** Geeraert BL, Lebel RM, Lebel C (2019). A multiparametric analysis of white matter maturation during late childhood and adolescence. DOI: 10.1002/hbm.24706. Reason for inclusion: Included because it provides a detailed multiparametric account of white-matter maturation during the target developmental window.
34. **[PubMed]** Corbett BA, Vandekar S, Muscatello RA, Tanguturi Y (2020). Pubertal Timing During Early Adolescence: Advanced Pubertal Onset in Females with Autism Spectrum Disorder. DOI: 10.1002/aur.2406. Reason for inclusion: Included as a developmental-timing extension because it examines sex-linked pubertal timing in an autistic adolescent sample.
35. **[PubMed]** Groenman AP, van der Oord S, Geurts HM (2024). Navigating adolescence: pubertal development in autism spectrum conditions and its relation to mental health. DOI: 10.1007/s00737-023-01414-0. Reason for inclusion: Included as a developmental-timing extension because it evaluates pubertal development patterns in autistic adolescents with sex-specific relevance.
36. **[PubMed]** Tsompanidis A, Hampton S, Aydin E, Allison C, Holt R, Baron-Cohen S (2023). Mini-puberty testosterone and infant autistic traits. DOI: 10.3389/fendo.2023.1126023. Reason for inclusion: Included because it addresses an early postnatal hormonal window that may inform later sex-related neurodevelopmental trajectories.
37. **[WoS unique]** Giedd, JN Castellanos, FX Rajapakse, JC Vaituzis, AC Rapoport, JL (1997). Sexual dimorphism of the developing human brain. PROGRESS IN NEURO-PSYCHOPHARMACOLOGY & BIOLOGICAL PSYCHIATRY. DOI: 10.1016/S0278-5846(97)00158-9. Reason for inclusion: Included because it is a foundational developmental MRI study directly characterizing sex-specific trajectories of brain anatomy during childhood and adolescence.
38. **[WoS unique]** Giedd, JN Blumenthal, J Jeffries, NO Rajapakse, JC Vaituzis, AC Liu, H Berry, YC Tobin, M Nelson, J Castellanos, FX (1999). Development of the human corpus callosum during childhood and adolescence: A longitudinal MRI study. PROGRESS IN NEURO-PSYCHOPHARMACOLOGY & BIOLOGICAL PSYCHIATRY. DOI: Not listed in screened export. Reason for inclusion: Included because it is a longitudinal MRI study of corpus callosum development that explicitly evaluates developmental change and possible sex differences during a core developmental window.
39. **[WoS unique]** De Bellis, MD Keshavan, MS Beers, SR Hall, J Frustaci, K Masalehdan, A Noll, J Boring, AM (2001). Sex differences in brain maturation during childhood and adolescence. CEREBRAL CORTEX. DOI: 10.1093/cercor/11.6.552. Reason for inclusion: Included because it directly examines age-by-sex differences in gray matter, white matter, and corpus callosal maturation in healthy youth.
40. **[WoS unique]** Killgore, WDS Oki, M Yurgelun-Todd, DA (2001). Sex-specific developmental changes in amygdala responses to affective faces. NEUROREPORT. DOI: 10.1097/00001756-200102120-00047. Reason for inclusion: Included because it tests sex-specific developmental change in affect-related prefrontal-amygdala circuitry during adolescence.
41. **[WoS unique]** Campbell, IG Darchia, N Khaw, WY Higgins, LM Feinberg, I (2005). Sleep EEG evidence of sex differences in adolescent brain maturation. SLEEP. DOI: 10.1093/sleep/28.5.637. Reason for inclusion: Included because it uses developmental sleep EEG markers to show earlier late-maturational change in girls than boys during adolescence.
42. **[WoS unique]** Schmithorst, VJ Holland, SK (2006). Functional MRI evidence for disparate developmental processes underlying intelligence in boys and girls. NEUROIMAGE. DOI: 10.1016/j.neuroimage.2006.01.010. Reason for inclusion: Included because it reports sex-by-age differences in functional activation/connectivity linked to intelligence across childhood and adolescence.
43. **[WoS unique]** MacMaster, FP Keshavan, M Mirza, Y Carrey, N Upadhyaya, AR El-Sheikh, R Buhagiar, CJ Taormina, SP Boyd, C Lynch, M Rose, M Ivey, J Moore, GJ Rosenberg, DR (2007). Development and sexual dimorphism of the pituitary gland. LIFE SCIENCES. DOI: 10.1016/j.lfs.2006.11.040.

Reason for inclusion: Included because it provides MRI evidence of age- and sex-related pituitary development during a pubertally relevant developmental period.

44. **[WoS unique]** Schmithorst, VJ Holland, SK (2007). Sex differences in the development of neuroanatomical functional connectivity underlying intelligence found using Bayesian connectivity analysis. NEUROIMAGE. DOI: 10.1016/j.neuroimage.2006.11.046. Reason for inclusion: Included because it addresses sexual dimorphism in the developmental relation between functional connectivity and intelligence in children.
45. **[WoS unique]** Neufang, S Specht, K Hausmann, M Güntürkün, O Herpertz-Dahlmann, B Fink, GR Konrad, K (2009). Sex Differences and the Impact of Steroid Hormones on the Developing Human Brain. CEREBRAL CORTEX. DOI: 10.1093/cercor/bhn100. Reason for inclusion: Included because it links pubertal stage and circulating steroid hormones with sex-differential gray matter development in youth.
46. **[WoS unique]** Barendse, MEA Swartz, JR Taylor, SL Fine, JR Shirtcliff, EA Yoon, L McMillan, SJ Tully, LM Guyer, AE (2024). Sex and pubertal variation in reward-related behavior and neural activation in early adolescents. DEVELOPMENTAL COGNITIVE NEUROSCIENCE. DOI: 10.1016/j.dcn.2024.101358. Reason for inclusion: Included because it tests how sex and pubertal markers jointly relate to reward behavior and neural processing in early adolescence.
47. **[WoS unique]** Duffy, KA Wiglesworth, A Roediger, DJ Island, E Mueller, BA Luciana, M Klimes-Dougan, B Cullen, KR Fiecas, MB (2025). Characterizing the effects of age, puberty, and sex on variability in resting-state functional connectivity in late childhood and early adolescence. NEUROIMAGE. DOI: 10.1016/j.neuroimage.2025.121238. Reason for inclusion: Included because it directly models the unique and overlapping roles of age, puberty, and sex in dynamic functional connectivity variability.
48. **[WoS unique]** Bon, GM Walther, J Comasco, E Derntl, B Kaufmann, T (2025). Longitudinal development of sex differences in the limbic system is associated with age, puberty and mental health. COMMUNICATIONS BIOLOGY. DOI: 10.1038/s42003-025-08866-3. Reason for inclusion: Included because it longitudinally examines how sex differences in limbic brain structure unfold with age and puberty and links those differences to mental health.

3.2 Flow B: Phenotypic expression (3 studies)

49. **[PubMed]** Greenberg DM, Warrier V, Abu-Akel A, Allison C, Gajos KZ, Reinecke K, et al. (2023). Sex and age differences in "theory of mind" across 57 countries using the English version of the "Reading the Mind in the Eyes" Test. DOI: 10.1073/pnas.2022385119. Reason for inclusion: Included because it directly addresses sex-related social-cognitive phenotype across development in a very large international youth sample.
50. **[PubMed]** Gemignani J, Gervain J (2024). Brain responses to repetition-based rule-learning do not exhibit sex differences: an aggregated analysis of infant fNIRS studies. DOI: 10.1038/s41598-024-53092-2. Reason for inclusion: Included because it tests early neurocognitive phenotype and explicit sex comparison in infancy.
51. **[PubMed]** Mestre MV, Samper P, Frías MD, Tur AM (2009). Are women more empathetic than men? A longitudinal study in adolescence. DOI: 10.1017/s1138741600001499. Reason for inclusion: Included because it provides longitudinal adolescent evidence on sex-related social-cognitive development.

3.3 Flow C: Clinical visibility in ASD/ADHD (25 studies)

52. **[PubMed]** Rynkiewicz A, Schuller B, Marchi E, Piana S, Camurri A, Lassalle A, et al. (2016). An investigation of the 'female camouflage effect' in autism using a computerized ADOS-2 and a test of sex/gender differences. DOI: 10.1186/s13229-016-0073-0. Reason for inclusion: Included because it directly examines female camouflage and measurement sensitivity in autism assessment.
53. **[PubMed]** Parish-Morris J, Liberman MY, Cieri C, Herrington JD, Yerys BE, Bateman L, et al. (2017). Linguistic camouflage in girls with autism spectrum disorder. DOI: 10.1186/s13229-017-0164-6. Reason for inclusion: Included because it shows how sex-linked language presentation may reduce clinical visibility in autistic girls.
54. **[PubMed]** Lundström S, Mårland C, Kuja-Halkola R, Anckarsäter H, Lichtenstein P, Gillberg C, et al. (2019). Assessing autism in females: The importance of a sex-specific comparison. DOI: 10.1016/j.psychres.2019.112566. Reason for inclusion: Included because it explicitly tests the need for sex-specific comparison when evaluating autism in females.

55. **[PubMed]** Yeung TS, Greene RK, Dick CC, Duvall SW (2024). Females evaluated for autism: characteristics and co-occurring and differential DSM-5 diagnoses. DOI: 10.1080/13854046.2023.2281709. Reason for inclusion: Included because it characterizes differential diagnostic profiles among females referred for autism evaluation.
56. **[PubMed]** Ratto AB, Kenworthy L, Yerys BE, Bascom J, Wieckowski AT, White SW, et al. (2018). What About the Girls? Sex-Based Differences in Autistic Traits and Adaptive Skills. DOI: 10.1007/s10803-017-3413-9. Reason for inclusion: Included because it addresses sex-based differences in autistic presentation relevant to recognition and underidentification.
57. **[PubMed]** Corbett BA, Schwartzman JM, Libsack EJ, Muscatello RA, Lerner MD, Simmons GL, et al. (2021). Camouflaging in Autism: Examining Sex-Based and Compensatory Models in Social Cognition and Communication. DOI: 10.1002/aur.2440. Reason for inclusion: Included because it directly evaluates sex-based and compensatory models of autistic camouflaging.
58. **[PubMed]** Song A, Cola M, Plate S, Petrulla V, Yankowitz L, Pandey J, et al. (2021). Natural language markers of social phenotype in girls with autism. DOI: 10.1111/jcpp.13348. Reason for inclusion: Included because it identifies female-typed social-language features that may influence clinical visibility.
59. **[PubMed]** Putnam OC, McFayden TC, Harrop C (2025). Sex Differences and Parent-Teacher Discrepancies in Reports of Autism Traits: Evidence for Camouflaging in a School Setting. DOI: 10.1007/s10803-024-06498-w. Reason for inclusion: Included because discrepant informant reports are directly relevant to school-based visibility and referral pathways.
60. **[PubMed]** Biederman J, Mick E, Faraone SV, Braaten E, Doyle A, Spencer T, et al. (2002). Influence of gender on attention deficit hyperactivity disorder in children referred to a psychiatric clinic. DOI: 10.1176/appi.ajp.159.1.36. Reason for inclusion: Included because it is a classic clinic-referred ADHD study on differential symptom presentation and recognition by gender.
61. **[PubMed]** Mowlem F, Agnew-Blais J, Taylor E, Asherson P (2019). Do different factors influence whether girls versus boys meet ADHD diagnostic criteria? Sex differences among children with high ADHD symptoms. DOI: 10.1016/j.psychres.2018.12.128. Reason for inclusion: Included because it directly tests sex differences in diagnostic-threshold pathways among children with high ADHD symptoms.
62. **[PubMed]** Mowlem FD, Rosenqvist MA, Martin J, Lichtenstein P, Asherson P, Larsson H (2019). Sex differences in predicting ADHD clinical diagnosis and pharmacological treatment. DOI: 10.1007/s00787-018-1211-3. Reason for inclusion: Included because it examines sex-differential transition from symptoms to diagnosis and treatment.
63. **[PubMed]** Madsen KB, Ravn MH, Arnfred J, Olsen J, Rask CU, Obel C (2018). Characteristics of undiagnosed children with parent-reported ADHD behaviour. DOI: 10.1007/s00787-017-1029-4. Reason for inclusion: Included because undiagnosed high-symptom cases are highly relevant to underrecognition and referral filtering.
64. **[PubMed]** Martínez-Jaime MM, Reyes-Morales H, Peyrot-Negrete I, Barrientos-Álvarez MS (2024). Access to early diagnosis for attention-deficit/hyperactivity disorder among children and adolescents in Mexico City at specialized mental health services. DOI: 10.1186/s12913-024-11022-y. Reason for inclusion: Included because it addresses barriers to early ADHD diagnosis in a real-world clinical service setting.
65. **[PubMed]** Ramtekkar UP, Reiersen AM, Todorov AA, Todd RD (2010). Sex and age differences in attention-deficit/hyperactivity disorder symptoms and diagnoses: implications for DSM-V and ICD-11. DOI: Not listed in the screened export. Reason for inclusion: Included because it directly addresses sex- and age-related differences in ADHD symptoms and diagnostic capture.
66. **[PubMed]** Gross IM, Gao Y, Lee MJ, Hipwell AE, Keenan K (2024). The ADHD Phenotype in Black and White Girls From Childhood to Adolescence: Results From the Community-Based Pittsburgh Girls Study. DOI: 10.1177/10870547231215281. Reason for inclusion: Included because it characterizes girls' ADHD presentation longitudinally in a community-based cohort, informing visibility beyond clinic samples.
67. **[WoS unique]** MCGEE, R FEEHAN, M (1991). ARE GIRLS WITH PROBLEMS OF ATTENTION UNDERRECOGNIZED. JOURNAL OF PSYCHOPATHOLOGY AND BEHAVIORAL ASSESSMENT. DOI: 10.1007/BF00960783. Reason for inclusion: Included because it is an early directly relevant paper explicitly questioning the underrecognition of girls with attention problems and arguing for sex-sensitive identification.
68. **[WoS unique]** BROWN, RT MADANSWAIN, A BALDWIN, K (1991). GENDER DIFFERENCES IN A CLINIC-REFERRED SAMPLE OF ATTENTION-DEFICIT-DISORDERED CHILDREN. CHILD PSYCHIATRY & HUMAN DEVELOPMENT. DOI: 10.1007/BF00707789. Reason for inclusion: Included because it examines sex differences in a clinic-referred ADHD sample and highlights later referral and broader impairment in girls.

69. **[WoS unique]** Sharp, WS Walter, JM Marsh, WL Ritchie, CF Hamburger, SD Castellanos, FX (1999). ADHD in girls: Clinical comparability of a research sample. JOURNAL OF THE AMERICAN ACADEMY OF CHILD AND ADOLESCENT PSYCHIATRY. DOI: 10.1097/00004583-199901000-00018. Reason for inclusion: Included because it directly addresses referral bias and sample ascertainment when identifying girls with ADHD.
70. **[WoS unique]** Biederman, J Faraone, SV Mick, E Williamson, S Wilens, TE Spencer, TJ Weber, W Jetton, J Kraus, I Pert, J Zallen, B (1999). Clinical correlates of ADHD in females: Findings from a large group of girls ascertained from pediatric and psychiatric referral sources. JOURNAL OF THE AMERICAN ACADEMY OF CHILD AND ADOLESCENT PSYCHIATRY. DOI: 10.1097/00004583-199908000-00012. Reason for inclusion: Included because it characterizes female ADHD in referred samples and is directly relevant to clinical visibility and underidentification.
71. **[WoS unique]** Sciutto, MJ Nolfi, CJ Bluhm, C (2004). Effects of child gender and symptom type on referrals for ADHD by elementary school teachers. JOURNAL OF EMOTIONAL AND BEHAVIORAL DISORDERS. DOI: 10.1177/10634266040120040501. Reason for inclusion: Included because it directly tests referral bias by showing that teacher referral decisions differ by child gender and symptom presentation.
72. **[WoS unique]** Tang, CH Chi, MH Hsieh, YT Lee, TI Tai, YC Lien, YJ Yang, YK Chen, PS (2022). Sex differences in the diagnosis of autism spectrum disorder and effects of comorbid mental retardation and attention-deficit disorder. JOURNAL OF THE FORMOSAN MEDICAL ASSOCIATION. DOI: 10.1016/j.jfma.2021.03.009. Reason for inclusion: Included because it directly examines sex-differential ASD diagnosis and shows how comorbid ADHD/MR alters diagnostic capture and age at diagnosis.
73. **[WoS unique]** Martin, J Rouquette, OY Langley, K Cooper, M Sayal, K Ford, TJ John, A Thapar, A (2026). Antecedents and outcomes of a later attention-deficit hyperactivity disorder (ADHD) diagnosis in females. BRITISH JOURNAL OF PSYCHIATRY. DOI: 10.1192/bjp.2026.10556. Reason for inclusion: Included because it directly examines delayed ADHD diagnosis in females and its adverse downstream outcomes.
74. **[WoS unique]** Maciver, D Roy, AS Johnston, L Boilson, M Curnow, E Johnstone-Cooke, V Rutherford, M (2026). Are we getting better at identifying and diagnosing neurodivergent girls and women? Insights into sex ratios and age of diagnosis from clinical population data in Scotland. AUTISM. DOI: 10.1177/13623613251383343. Reason for inclusion: Included because it directly evaluates sex ratios and age at referral/diagnosis, with explicit evidence of later referral and diagnosis in females.
75. **[WoS unique]** Ntini, I Nilsson, K Ramklint, M Aila-Gustafsson, S Sonnby, K (2026). Influence of sex and depressive symptoms on diagnostic delay of attention deficit hyperactivity disorder in adolescent psychiatric patients. NORDIC JOURNAL OF PSYCHIATRY. DOI: 10.1080/08039488.2025.2588759. Reason for inclusion: Included because it directly quantifies diagnostic delay in girls with ADHD and identifies depressive symptoms as an interference factor.
76. **[WoS unique]** Ercan, ES Tahillioglu, A Tufan, AE Bilaç, Ö (2025). Teachers predict ADHD more accurately than parents: findings from a large epidemiological survey. NORDIC JOURNAL OF PSYCHIATRY. DOI: 10.1080/08039488.2025.2508417. Reason for inclusion: Included because it is directly relevant to informant-based detection and shows that diagnostic accuracy varies by informant and child gender.

4. Deduplicated master list of included reviews, meta-analyses, and key syntheses

4.1 Flow A: Developmental timing reviews and syntheses (9 studies)

77. **[PubMed]** Lenroot RK, Giedd JN (2010). Sex differences in the adolescent brain. DOI: 10.1016/j.bandc.2009.10.008. Reason for inclusion: Included because it is a directly relevant review synthesizing sex differences in adolescent brain development.
78. **[PubMed]** Piekarski DJ, Colich NL, Ho TC (2023). The effects of puberty and sex on adolescent white matter development: A systematic review. DOI: 10.1016/j.dcn.2023.101214. Reason for inclusion: Included because it is a directly relevant systematic review for puberty-, sex-, and white-matter-related developmental timing.
79. **[PubMed]** Cousminer DL, Widén E, Palmert MR (2016). The genetics of pubertal timing in the general population: recent advances and evidence for sex-specificity. DOI: 10.1097/MED.0000000000000213. Reason for inclusion: Included because it is a targeted review of sex-specific aspects of pubertal timing.

80. **[PubMed]** Ullsperger JM, Nikolas MA (2017). A meta-analytic review of the association between pubertal timing and psychopathology in adolescence: Are there sex differences in risk? 333. DOI: 10.1037/bul0000106. Reason for inclusion: Included because it meta-analytically evaluates pubertal timing and explicitly tests sex-differential risk.
81. **[PubMed]** Schulz KM, Molenda-Figueira HA, Sisk CL (2009). Back to the future: The organizational-activational hypothesis adapted to puberty and adolescence. DOI: 10.1016/j.yhbeh.2009.03.010. Reason for inclusion: Included because it is a foundational theoretical review on puberty-related sexual differentiation during adolescence.
82. **[PubMed]** Kuhn C, Johnson M, Thomae A, Luo B, Simon SA, Zhou G, et al. (2010). The emergence of gonadal hormone influences on dopaminergic function during puberty. DOI: 10.1016/j.yhbeh.2009.10.015. Reason for inclusion: Included because it reviews puberty-linked gonadal-hormone mechanisms relevant to sex-differential neurodevelopment.
83. **[PubMed]** Barendse MEA, Lara GA, Guyer AE, Swartz JR, Taylor SL, Shirtcliff EA, et al. (2023). Sex and pubertal influences on the neurodevelopmental underpinnings of schizophrenia: A case for longitudinal research on adolescents. DOI: 10.1016/j.schres.2022.12.011. Reason for inclusion: Included because it is a review article on sex/puberty effects in adolescent neurodevelopment.
84. **[WoS unique]** Giedd, JN Raznahan, A Mills, KL Lenroot, RK (2012). Review: magnetic resonance imaging of male/female differences in human adolescent brain anatomy. BIOLOGY OF SEX DIFFERENCES. DOI: 10.1186/2042-6410-3-19. Reason for inclusion: Included because it is a directly relevant synthesis of MRI evidence on male/female differences in adolescent brain anatomy.
85. **[WoS unique]** Ladouceur, CD Peper, JS Crone, EA Dahl, RE (2012). White matter development in adolescence: The influence of puberty and implications for affective disorders. DEVELOPMENTAL COGNITIVE NEUROSCIENCE. DOI: 10.1016/j.dcn.2011.06.002. Reason for inclusion: Included because it is a targeted review of puberty-linked white matter development that critically discusses sex differences and developmental timing.

4.2 Flow B: Phenotypic expression reviews and syntheses (14 studies)

86. **[PubMed]** Adani S, Cepanec M (2019). Sex differences in early communication development: behavioral and neurobiological indicators of more vulnerable communication system development in boys. DOI: 10.3325/cmj.2019.60.141. Reason for inclusion: Included because it is a targeted review of early communication phenotype with explicit sex-related developmental framing.
87. **[PubMed]** Etchell A, Adhikari A, Weinberg LS, Choo AL, Garnett EO, Chow HM, et al. (2018). A systematic literature review of sex differences in childhood language and brain development. DOI: 10.1016/j.neuropsychologia.2018.04.011. Reason for inclusion: Included because it directly synthesizes language and brain-development evidence in childhood.
88. **[PubMed]** Chaplin TM, Aldao A (2013). Gender differences in emotion expression in children: a meta-analytic review. DOI: 10.1037/a0030737. Reason for inclusion: Included because it is a core meta-analysis for emotion-expression phenotype in childhood.
89. **[PubMed]** Thompson AE, Voyer D (2014). Sex differences in the ability to recognise non-verbal displays of emotion: a meta-analysis. DOI: 10.1080/02699931.2013.875889. Reason for inclusion: Included because it is a core meta-analysis for emotion-recognition phenotype.
90. **[PubMed]** Margoni F, Block K, Hamlin K, Zmyj N, Schmader T (2023). Meta-analytic evidence against sex differences in infants' and toddlers' preference for prosocial agents. DOI: 10.1037/dev0001421. Reason for inclusion: Included because it directly evaluates early prosocial phenotype through meta-analysis.
91. **[PubMed]** Xiao SX, Hashi EC, Korous KM, Eisenberg N (2019). Gender differences across multiple types of prosocial behavior in adolescence: A meta-analysis of the prosocial tendency measure-revised (PTM-R). DOI: 10.1016/j.adolescence.2019.09.003. Reason for inclusion: Included because it meta-analyzes adolescent prosocial phenotype across multiple subtypes.
92. **[PubMed]** Wood-Downie H, Wong B, Kovshoff H, Cortese S, Hadwin JA (2021). Research Review: A systematic review and meta-analysis of sex/gender differences in social interaction and communication in autistic and nonautistic children and adolescents. DOI: 10.1111/jcpp.13337. Reason for inclusion: Included because it directly synthesizes sex/gender differences in social-communication phenotype in youth.
93. **[PubMed]** Edwards H, Wright S, Sargeant C, Cortese S, Wood-Downie H (2024). Research Review: A systematic review and meta-analysis of sex differences in narrow constructs of restricted and repetitive behaviours and interests in autistic children, adolescents, and adults. DOI: 10.1111/jcpp.13855. Reason for inclusion: Included because it synthesizes a clinically relevant autistic phenotype with direct sex-difference implications.

94. **[PubMed]** Granocchio E, De Salvatore M, Bonanomi E, Sarti D (2023). Sex-related differences in reading achievement. DOI: 10.1002/jnr.24913. Reason for inclusion: Included because it is a targeted review of sex-related differences in reading phenotype and developmental emergence.
95. **[WoS unique]** McClure, EB (2000). A meta-analytic review of sex differences in facial expression processing and their development in infants, children, and adolescents. PSYCHOLOGICAL BULLETIN. DOI: 10.1037/0033-2909.126.3.424. Reason for inclusion: Included because it is a directly relevant meta-analysis of developmental sex differences in facial expression processing.
96. **[WoS unique]** Leaper, C Smith, TE (2004). A meta-analytic review of gender variations in children's language use: Talkativeness, affiliative speech, and assertive speech. DEVELOPMENTAL PSYCHOLOGY. DOI: 10.1037/0012-1649.40.6.993. Reason for inclusion: Included because it is a core meta-analysis of sex/gender differences in children's language phenotype.
97. **[WoS unique]** Herba, C Phillips, M (2004). Annotation: Development of facial expression recognition from childhood to adolescence: behavioural and neurological perspectives. JOURNAL OF CHILD PSYCHOLOGY AND PSYCHIATRY. DOI: 10.1111/j.1469-7610.2004.00316.x. Reason for inclusion: Included because it is a focused review of the developmental emergence of facial-expression recognition and associated neural systems, including sex-related considerations.
98. **[WoS unique]** Silverman, IW (2021). Gender differences in inhibitory control as assessed on simple delay tasks in early childhood: A meta-analysis. INTERNATIONAL JOURNAL OF BEHAVIORAL DEVELOPMENT. DOI: 10.1177/01650254211020385. Reason for inclusion: Included because it is a directly relevant meta-analysis of an executive-function phenotype in early childhood.
99. **[WoS unique]** Doidge, JL Flora, DB Toplak, ME (2021). A Meta-Analytic Review of Sex Differences on Delay of Gratification and Temporal Discounting Tasks in ADHD and Typically Developing Samples. JOURNAL OF ATTENTION DISORDERS. DOI: 10.1177/1087054718815588. Reason for inclusion: Included because it synthesizes sex differences in delay-related executive/reward decision-making across ADHD and typically developing samples.

4.3 Flow C: Clinical visibility reviews and syntheses (11 studies)

100. **[PubMed]** Kreiser NL, White SW (2014). ASD in females: are we overstating the gender difference in diagnosis? 2076. DOI: 10.1007/s10567-013-0148-9. Reason for inclusion: Included because it is a key review on underrecognition and diagnostic bias in females with ASD.
101. **[PubMed]** Cook J, Hull L, Crane L, Mandy W (2021). Camouflaging in autism: A systematic review. DOI: 10.1016/j.cpr.2021.102080. Reason for inclusion: Included because it is a central systematic review on camouflaging and clinical visibility in autism.
102. **[PubMed]** Wood-Downie H, Wong B, Kovshoff H, Mandy W, Hull L, Hadwin JA (2021). Sex/Gender Differences in Camouflaging in Children and Adolescents with Autism. DOI: 10.1007/s10803-020-04615-z. Reason for inclusion: Included because it specifically synthesizes child/adolescent evidence on sex/gender differences in camouflaging.
103. **[PubMed]** Gaub M, Carlson CL (1997). Gender differences in ADHD: a meta-analysis and critical review. DOI: 10.1097/00004583-199708000-00011. Reason for inclusion: Included because it is a foundational meta-analytic review on sex differences in ADHD presentation.
104. **[PubMed]** Loyer Carbonneau M, Demers M, Bigras M, Guay MC (2021). Meta-Analysis of Sex Differences in ADHD Symptoms and Associated Cognitive Deficits. DOI: 10.1177/1087054720923736. Reason for inclusion: Included because it directly synthesizes sex differences in ADHD symptoms and associated cognitive deficits.
105. **[PubMed]** Kok FM, Groen Y, Fuermaier AB, Tucha O (2016). Problematic Peer Functioning in Girls with ADHD: A Systematic Literature Review. DOI: 10.1371/journal.pone.0165119. Reason for inclusion: Included because it reviews a female-typed ADHD social phenotype with implications for lower visibility.
106. **[PubMed]** Dimitri D, Delia G, Cavallo F, Varini M, Fioretto F (2025). Sex differences in children and adolescents with attention-deficit/hyperactivity disorder: a literature review. DOI: 10.3389/frcha.2025.1582502. Reason for inclusion: Included because it is a recent narrative review focused specifically on sex differences in child/adolescent ADHD.
107. **[WoS unique]** Babinski, DE (2024). Sex Differences in ADHD: Review and Priorities for Future Research. CURRENT PSYCHIATRY REPORTS. DOI: 10.1007/s11920-024-01492-6. Reason for inclusion: Included because it is a recent targeted review emphasizing identification, sex/gender issues, and future priorities in ADHD research.

108. **[WoS unique]** Rucklidge, JJ (2010). Gender Differences in Attention-Deficit/Hyperactivity Disorder. PSYCHIATRIC CLINICS OF NORTH AMERICA. DOI: 10.1016/j.psc.2010.01.006. Reason for inclusion: Included because it is a directly relevant review of gender differences in ADHD presentation and referral bias across the lifespan.
109. **[WoS unique]** Yu, YY Wyman, A Faulk, CJ Fulop, LJ Greenberg, RL Benecke, RM Steinbeck, LK Foy, J Kim, C Emory, GO Fox, E Storch, EA Zampella, CJ Yerys, BE Schultz, RT Parish-Morris, J Herrington, JD Clements, CC (2026). The Under-Identification of Autism in Females: A Review and Analysis of Sex-Based Scoring Differences Observed in Autism Diagnostic Observation Schedule (ADOS) Module 3. JOURNAL OF AUTISM AND DEVELOPMENTAL DISORDERS. DOI: 10.1007/s10803-026-07253-z. Reason for inclusion: Included because it directly addresses under-identification of autism in females and sex-based scoring issues in a core diagnostic instrument.
110. **[WoS unique]** Minutoli, R Marraffa, C Failla, C Pioggia, G Marino, F (2026). Female gender and autism: underdiagnosis and misdiagnosis - clinical and scientific urgency. FRONTIERS IN PSYCHIATRY. DOI: 10.3389/fpsyt.2025.1704579. Reason for inclusion: Included because it is a directly relevant review focused on female underdiagnosis and misdiagnosis in autism.

Supplementary Table S3. Characteristics of included primary empirical studies (n = 76)

Study ID	Flow	Citation	Source	Developmental window / context	Broad design / modality	Primary domain	Main contribution / evidence role
AE01	A	Ingalhalikar et al. (2014)	PubMed	Youth / mixed developmental window; General developmental sample	Neuroimaging / neurophysiology study	Functional connectivity / network organization	It directly characterizes sex-related differences in large-scale structural brain connectivity during youth development.
AE02	A	Beck et al. (2023)	PubMed	Adolescence / pubertal transition; General developmental sample	Longitudinal / developmental trajectory study	Cortical / global brain development	It explicitly models puberty-related brain maturation and compares male and female developmental trajectories.
AE03	A	Tomasi et al. (2023)	PubMed	Childhood; General developmental sample	Neuroimaging / neurophysiology study	Functional connectivity / network organization	It links sex-related differences in childhood brain connectivity to cognitive performance in a large developmental cohort.
AE04	A	Fish et al. (2020)	PubMed	Childhood to adolescence; General developmental sample	Longitudinal / developmental trajectory study	Limbic / subcortical development	It maps sex-biased developmental trajectories in limbic morphology across childhood and adolescence.
AE05	A	Sanders et al. (2023)	PubMed	Childhood; General developmental sample	Longitudinal / developmental trajectory study	Functional connectivity / network organization	It reports age-related network development and tests whether sex moderates those trajectories.
AE06	A	Shanmugan et al. (2022)	PubMed	Youth / mixed developmental window; General developmental sample	Primary empirical study	Functional connectivity / network organization	It addresses sex-differential organization of functional association networks in a youth sample.
AE07	A	Forde et al. (2017)	PubMed	Adolescence / pubertal transition; General developmental sample	Neuroimaging / neurophysiology study	Cortical / global brain development	It examines age and sex effects on multiple cortical morphometric indices during adolescence.
AE08	A	Lynch et al. (2019)	PubMed	Childhood to adolescence; General developmental sample	Primary empirical study	Limbic / subcortical development	It evaluates age-related hippocampal maturation and sex interactions in a large developmental sample.
AE09	A	Mills et al. (2021)	PubMed	Late childhood; General developmental sample	Neuroimaging / neurophysiology study	Developmental timing	It quantifies developmental variability and sex differences in structural brain change.
AE10	A	Satterthwaite et al. (2014)	PubMed	Adolescence / pubertal transition; General developmental sample	Neuroimaging / neurophysiology study	Limbic / subcortical development	It directly tests sex-by-puberty effects on hippocampal development.
AE11	A	Whittle et al. (2020)	PubMed	Childhood; General developmental sample	Longitudinal / developmental trajectory study	Limbic / subcortical development	It links pubertal hormones with sex-specific neuroanatomical trajectories.
AE12	A	Picci et al. (2023)	PubMed	NR in evidence-base note; General developmental sample	Neuroimaging / neurophysiology study	Cortical / global brain development	It assesses hormone-linked sex-differential changes in cortical function during development.

Study ID	Flow	Citation	Source	Developmental window / context	Broad design / modality	Primary domain	Main contribution / evidence role
AE13	A	Frere et al. (2020)	PubMed	Adolescence / pubertal transition; General developmental sample	Primary empirical study	Limbic / subcortical development	It examines sex-related limbic maturation in adolescence and relates it to emotion regulation.
AE14	A	Corrigan et al. (2024)	PubMed	Adolescence / pubertal transition; General developmental sample	Neuroimaging / neurophysiology study	Cortical / global brain development	It addresses sex-differential maturation patterns in an adolescent brain-development context.
AE15	A	Ott et al. (2021)	PubMed	Adolescence / pubertal transition; General developmental sample	Neuroimaging / neurophysiology study	Cortical / global brain development	It provides age- and sex-sensitive developmental neurophysiology during the transition to adolescence.
AE16	A	Schmithorst et al. (2008)	PubMed	NR in evidence-base note; General child/adolescent comparison sample	Neuroimaging / neurophysiology study	White matter / structural maturation	It is a foundational developmental dti study of sex-related white-matter maturation.
AE17	A	Bava et al. (2011)	PubMed	Adolescence / pubertal transition; General developmental sample	Neuroimaging / neurophysiology study	White matter / structural maturation	It further characterizes sex-related developmental white-matter architecture in adolescence.
AE18	A	Seunarine et al. (2016)	PubMed	NR in evidence-base note; General developmental sample	Longitudinal / developmental trajectory study	White matter / structural maturation	It directly models sexually dimorphic white-matter developmental trajectories.
AE19	A	Mutlu et al. (2013)	PubMed	NR in evidence-base note; General developmental sample	Neuroimaging / neurophysiology study	Cortical / global brain development	It examines sex-related developmental changes in cortical thickness and gyrification.
AE20	A	Mills et al. (2016)	PubMed	Childhood; General developmental sample	Longitudinal / developmental trajectory study	Developmental timing	It synthesizes longitudinal structural-brain development across cohorts and is central to developmental-timing inference.
AE21	A	Herting et al. (2015)	PubMed	Adolescence / pubertal transition; General developmental sample	Longitudinal / developmental trajectory study	Cortical / global brain development	It specifically links pubertal maturation to longitudinal cortical development.
AE22	A	Herting et al. (2018)	PubMed	Adolescence / pubertal transition; General developmental sample	Longitudinal / developmental trajectory study	Cortical / global brain development	It provides multisample longitudinal evidence on sex-related subcortical developmental change.
AE23	A	Peper et al. (2009)	PubMed	Adolescence / pubertal transition; General child/adolescent comparison sample	Neuroimaging / neurophysiology study	Cortical / global brain development	It connects pubertal hormones to sex-related brain-structure variation during adolescence.
AE24	A	Nguyen et al. (2013)	PubMed	Childhood to adolescence; General developmental sample	Neuroimaging / neurophysiology study	Cortical / global brain development	It directly models testosterone-associated cortical maturation across development.
AE25	A	Herting et al. (2017)	PubMed	Adolescence / pubertal transition; General developmental sample	Longitudinal / developmental trajectory study	White matter / structural maturation	It longitudinally examines puberty-linked white-matter maturation with explicit sex relevance.
AE26	A	Herting et al. (2012)	PubMed	Adolescence / pubertal transition; General developmental sample	Neuroimaging / neurophysiology study	White matter / structural maturation	It jointly evaluates sex, puberty, and hormone effects on adolescent white matter.
AE27	A	Wierenga et al. (2018)	PubMed	NR in evidence-base note; General child/adolescent comparison sample	Neuroimaging / neurophysiology study	Cortical / global brain development	It directly addresses sex-related variability as a developmental-timing phenomenon.
AE28	A	Pangelinan et al. (2016)	PubMed	Adolescence / pubertal transition; General developmental sample	Primary empirical study	Puberty / hormonal timing	It addresses hormone-related maturation during adolescence within the developmental-timing framework.
AE29	A	Campbell et al. (2012)	PubMed	Adolescence / pubertal transition; General developmental sample	Neuroimaging / neurophysiology study	Cortical / global brain development	It uses sleep eeg as an index of adolescent brain maturation and examines sex and puberty jointly.
AE30	A	Lawrence et al. (2023)	PubMed	Late childhood; General developmental sample	Neuroimaging / neurophysiology study	White matter / structural maturation	It reports large-sample late-childhood white-matter sex differences relevant to developmental timing.
AE31	A	Yang et al. (2021)	PubMed	Childhood; General developmental sample	Neuroimaging / neurophysiology study	Developmental timing	It uses multimodal developmental neuroimaging to characterize sexual dimorphism in childhood brain development.

Study ID	Flow	Citation	Source	Developmental window / context	Broad design / modality	Primary domain	Main contribution / evidence role
AE32	A	Kurth et al. (2024)	PubMed	Childhood to adolescence; General developmental sample	Neuroimaging / neurophysiology study	Cortical / global brain development	It examines age- and sex-related asymmetry during neurodevelopment in a large youth sample.
AE33	A	Geeraert et al. (2019)	PubMed	Late childhood to adolescence; General developmental sample	Neuroimaging / neurophysiology study	White matter / structural maturation	It provides a detailed multiparametric account of white-matter maturation during the target developmental window.
AE34	A	Corbett et al. (2020)	PubMed	Early adolescence; Autism-related / autistic sample or autism-focused context	Primary empirical study	Developmental timing	Included as a developmental-timing extension because it examines sex-linked pubertal timing in an autistic adolescent sample.
AE35	A	Groenman et al. (2024)	PubMed	Adolescence / pubertal transition; Autism-related / autistic sample or autism-focused context	Primary empirical study	Developmental timing	Included as a developmental-timing extension because it evaluates pubertal development patterns in autistic adolescents with sex-specific relevance.
AE36	A	Tsompanidis et al. (2023)	PubMed	Infancy / early development; Autism-related / autistic sample or autism-focused context	Longitudinal / developmental trajectory study	Puberty / hormonal timing	It addresses an early postnatal hormonal window that may inform later sex-related neurodevelopmental trajectories.
AE37	A	Giedd et al. (1997)	WoS unique	Childhood to adolescence; General developmental sample	Longitudinal / developmental trajectory study	Developmental timing	It is a foundational developmental mri study directly characterizing sex-specific trajectories of brain anatomy during childhood.
AE38	A	Giedd et al. (1999)	WoS unique	Childhood to adolescence; General developmental sample	Longitudinal / developmental trajectory study	Developmental timing	It is a longitudinal mri study of corpus callosum development that explicitly evaluates developmental change.
AE39	A	De et al. (2001)	WoS unique	Childhood to adolescence; General developmental sample	Neuroimaging / neurophysiology study	White matter / structural maturation	It directly examines age-by-sex differences in gray matter, white matter, and corpus callosal maturation in healthy youth.
AE40	A	Killgore et al. (2001)	WoS unique	Adolescence / pubertal transition; General developmental sample	Primary empirical study	Limbic / subcortical development	It tests sex-specific developmental change in affect-related prefrontal-amygdala circuitry during adolescence.
AE41	A	Campbell et al. (2005)	WoS unique	Adolescence / pubertal transition; General child/adolescent comparison sample	Neuroimaging / neurophysiology study	Cortical / global brain development	It uses developmental sleep eeg markers to show earlier late-maturational change in girls than boys during adolescence.
AE42	A	Schmithorst et al. (2006)	WoS unique	Childhood to adolescence; General child/adolescent comparison sample	Neuroimaging / neurophysiology study	Functional connectivity / network organization	It reports sex-by-age differences in functional activation/connectivity linked to intelligence across childhood.
AE43	A	MacMaster et al. (2007)	WoS unique	Adolescence / pubertal transition; General developmental sample	Neuroimaging / neurophysiology study	Limbic / subcortical development	It provides mri evidence of age- and sex-related pituitary development during a pubertally relevant developmental period.
AE44	A	Schmithorst et al. (2007)	WoS unique	NR in evidence-base note; General developmental sample	Neuroimaging / neurophysiology study	Functional connectivity / network organization	It addresses sexual dimorphism in the developmental relation between functional connectivity.
AE45	A	Neufang et al. (2009)	WoS unique	Adolescence / pubertal transition; General developmental sample	Neuroimaging / neurophysiology study	Puberty / hormonal timing	It links pubertal stage and circulating steroid hormones with sex-differential gray matter development in youth.
AE46	A	Barendse et al. (2024)	WoS unique	Early adolescence; General developmental sample	Primary empirical study	Developmental timing	It tests how sex and pubertal markers jointly relate to reward behavior and neural processing in early adolescence.
AE47	A	Duffy et al. (2025)	WoS unique	Early adolescence; General developmental sample	Neuroimaging / neurophysiology study	Functional connectivity / network organization	It directly models the unique and overlapping roles of age, puberty, and sex in dynamic functional connectivity variability.

Study ID	Flow	Citation	Source	Developmental window / context	Broad design / modality	Primary domain	Main contribution / evidence role
AE48	A	Bon et al. (2025)	WoS unique	Adolescence / pubertal transition; General developmental sample	Longitudinal / developmental trajectory study	Limbic / subcortical development	It longitudinally examines how sex differences in limbic brain structure unfold with age.
BE01	B	Greenberg et al. (2023)	PubMed	Youth / mixed developmental window; General developmental sample	Primary empirical study	Language / communication	It directly addresses sex-related social-cognitive phenotype across development in a very large international youth sample.
BE02	B	Gemignani et al. (2024)	PubMed	Infancy / early development; General developmental sample	Neuroimaging / neurophysiology study	Phenotypic expression	It tests early neurocognitive phenotype and explicit sex comparison in infancy.
BE03	B	Mestre et al. (2009)	PubMed	Adolescence / pubertal transition; General developmental sample	Longitudinal / developmental trajectory study	Social cognition / empathy / prosociality	It provides longitudinal adolescent evidence on sex-related social-cognitive development.
CE01	C	Rynkiewicz et al. (2016)	PubMed	NR in evidence-base note; Autism-related / autistic sample or autism-focused context	Assessment / diagnostic / referral study	ASD clinical visibility / camouflaging	It directly examines female camouflage and measurement sensitivity in autism assessment.
CE02	C	Parish-Morris et al. (2017)	PubMed	NR in evidence-base note; Autism-related / autistic sample or autism-focused context	Primary empirical study	ASD clinical visibility / camouflaging	It shows how sex-linked language presentation may reduce clinical visibility in autistic girls.
CE03	C	Lundström et al. (2019)	PubMed	NR in evidence-base note; Autism-related / autistic sample or autism-focused context	Primary empirical study	ASD clinical visibility / camouflaging	It explicitly tests the need for sex-specific comparison when evaluating autism in females.
CE04	C	Yeung et al. (2024)	PubMed	NR in evidence-base note; Autism-related / autistic sample or autism-focused context	Primary empirical study	ASD clinical visibility / camouflaging	It characterizes differential diagnostic profiles among females referred for autism evaluation.
CE05	C	Ratto et al. (2018)	PubMed	NR in evidence-base note; Autism-related / autistic sample or autism-focused context	Primary empirical study	Clinical visibility	It addresses sex-based differences in autistic presentation relevant to recognition and underidentification.
CE06	C	Corbett et al. (2021)	PubMed	NR in evidence-base note; Autism-related / autistic sample or autism-focused context	Primary empirical study	ASD clinical visibility / camouflaging	It directly evaluates sex-based and compensatory models of autistic camouflaging.
CE07	C	Song et al. (2021)	PubMed	NR in evidence-base note; Autism-related / autistic sample or autism-focused context	Primary empirical study	ASD clinical visibility / camouflaging	It identifies female-typed social-language features that may influence clinical visibility.
CE08	C	Putnam et al. (2025)	PubMed	NR in evidence-base note; Autism-related / autistic sample or autism-focused context	Assessment / diagnostic / referral study	ASD clinical visibility / camouflaging	Discrepant informant reports are directly relevant to school-based visibility and referral pathways.
CE09	C	Biederman et al. (2002)	PubMed	NR in evidence-base note; ADHD-related / attention problems context	Primary empirical study	ADHD clinical visibility / referral and diagnosis	It is a classic clinic-referred adhd study on differential symptom presentation and recognition by gender.
CE10	C	Mowlem et al. (2019)	PubMed	NR in evidence-base note; ADHD-related / attention problems context	Primary empirical study	ADHD clinical visibility / referral and diagnosis	It directly tests sex differences in diagnostic-threshold pathways among children with high adhd symptoms.
CE11	C	Mowlem et al. (2019)	PubMed	NR in evidence-base note; ADHD-related / attention problems context	Assessment / diagnostic / referral study	ADHD clinical visibility / referral and diagnosis	It examines sex-differential transition from symptoms to diagnosis and treatment.
CE12	C	Madsen et al. (2018)	PubMed	NR in evidence-base note; ADHD-related / attention problems context	Assessment / diagnostic / referral study	ADHD clinical visibility / referral and diagnosis	Undiagnosed high-symptom cases are highly relevant to underrecognition and referral filtering.
CE13	C	Martínez-Jaime et al. (2024)	PubMed	Childhood to adolescence; ADHD-related / attention problems context	Assessment / diagnostic / referral study	ADHD clinical visibility / referral and diagnosis	It addresses barriers to early adhd diagnosis in a real-world clinical service setting.

Study ID	Flow	Citation	Source	Developmental window / context	Broad design / modality	Primary domain	Main contribution / evidence role
CE14	C	Ramtekkar et al. (2010)	PubMed	NR in evidence-base note; ADHD-related / attention problems context	Primary empirical study	ADHD clinical visibility / referral and diagnosis	It directly addresses sex- and age-related differences in adhd symptoms and diagnostic capture.
CE15	C	Gross et al. (2024)	PubMed	Childhood; ADHD-related / attention problems context	Longitudinal / developmental trajectory study	ADHD clinical visibility / referral and diagnosis	It characterizes girls' adhd presentation longitudinally in a community-based cohort.
CE16	C	MCGEE et al. (1991)	WoS unique	NR in evidence-base note; Clinical visibility / referral or assessment context	Assessment / diagnostic / referral study	Clinical visibility	It is an early directly relevant paper explicitly questioning the underrecognition of girls with attention problems.
CE17	C	BROWN et al. (1991)	WoS unique	NR in evidence-base note; ADHD-related / attention problems context	Assessment / diagnostic / referral study	ADHD clinical visibility / referral and diagnosis	It examines sex differences in a clinic-referred adhd sample and highlights later referral.
CE18	C	Sharp et al. (1999)	WoS unique	Adolescence / pubertal transition; ADHD-related / attention problems context	Assessment / diagnostic / referral study	ADHD clinical visibility / referral and diagnosis	It directly addresses referral bias and sample ascertainment when identifying girls with adhd.
CE19	C	Biederman et al. (1999)	WoS unique	Adolescence / pubertal transition; ADHD-related / attention problems context	Assessment / diagnostic / referral study	ADHD clinical visibility / referral and diagnosis	It characterizes female adhd in referred samples and is directly relevant to clinical visibility and underidentification.
CE20	C	Sciutto et al. (2004)	WoS unique	NR in evidence-base note; ADHD-related / attention problems context	Assessment / diagnostic / referral study	ADHD clinical visibility / referral and diagnosis	It directly tests referral bias by showing that teacher referral decisions differ by child gender.
CE21	C	Tang et al. (2022)	WoS unique	NR in evidence-base note; Autism-related / autistic sample or autism-focused context	Assessment / diagnostic / referral study	ASD clinical visibility / camouflaging	It directly examines sex-differential asd diagnosis and shows how comorbid adhd/mr alters diagnostic capture.
CE22	C	Martin et al. (2026)	WoS unique	NR in evidence-base note; ADHD-related / attention problems context	Assessment / diagnostic / referral study	ADHD clinical visibility / referral and diagnosis	It directly examines delayed adhd diagnosis in females and its adverse downstream outcomes.
CE23	C	Maciver et al. (2026)	WoS unique	NR in evidence-base note; Autism-related / autistic sample or autism-focused context	Assessment / diagnostic / referral study	ASD clinical visibility / camouflaging	It directly evaluates sex ratios and age at referral/diagnosis, with explicit evidence of later referral.
CE24	C	Ntini et al. (2026)	WoS unique	Adolescence / pubertal transition; ADHD-related / attention problems context	Primary empirical study	ADHD clinical visibility / referral and diagnosis	It directly quantifies diagnostic delay in girls with adhd and identifies depressive symptoms as an interference factor.
CE25	C	Ercan et al. (2025)	WoS unique	NR in evidence-base note; ADHD-related / attention problems context	Survey / epidemiological study	ADHD clinical visibility / referral and diagnosis	It is directly relevant to informant-based detection and shows that diagnostic accuracy varies by informant.

Supplementary Table S4. Characteristics of included review and synthesis sources (n = 34)

Review ID	Flow	Citation	Review type	Scope / developmental window	Primary domain	Main role in manuscript	Why retained / key contribution
AR01	A	Lenroot et al. (2010)	Narrative / targeted review	Adolescence / pubertal transition	Developmental timing	Foundation / synthesis support	It is a directly relevant review synthesizing sex differences in adolescent brain development.
AR02	A	Piekarski et al. (2023)	Systematic review	Adolescence / pubertal transition	White matter / structural maturation	Foundation / synthesis support	It is a directly relevant systematic review for puberty-, sex-, and white-matter-related developmental timing.
AR03	A	Cousminer et al. (2016)	Narrative / targeted review	Adolescence / pubertal transition	Developmental timing	Foundation / synthesis support	It is a targeted review of sex-specific aspects of pubertal timing.
AR04	A	Ullsperger et al. (2017)	Meta-analysis / meta-analytic review	Adolescence / pubertal transition	Developmental timing	Foundation / synthesis support	It meta-analytically evaluates pubertal timing and explicitly tests sex-differential risk.
AR05	A	Schulz et al. (2009)	Narrative / targeted review	Adolescence / pubertal transition	Puberty / hormonal timing	Foundation / synthesis support	It is a foundational theoretical review on puberty-related sexual differentiation during adolescence.
AR06	A	Kuhn et al. (2010)	Narrative / targeted review	Adolescence / pubertal transition	Puberty / hormonal timing	Foundation / synthesis support	It reviews puberty-linked gonadal-hormone mechanisms relevant to sex-differential neurodevelopment.
AR07	A	Barendse et al. (2023)	Narrative / targeted review	Adolescence / pubertal transition	Puberty / hormonal timing	Foundation / synthesis support	It is a review article on sex/puberty effects in adolescent neurodevelopment.
AR08	A	Giedd et al. (2012)	Narrative / targeted review	Adolescence / pubertal transition	Developmental timing	Foundation / synthesis support	It is a directly relevant synthesis of mri evidence on male/female differences in adolescent brain anatomy.
AR09	A	Ladouceur et al. (2012)	Narrative / targeted review	Adolescence / pubertal transition	White matter / structural maturation	Foundation / synthesis support	It is a targeted review of puberty-linked white matter development that critically discusses sex differences.
BR01	B	Adani et al. (2019)	Narrative / targeted review	NR in evidence-base note	Language / communication	Foundation / synthesis support	It is a targeted review of early communication phenotype with explicit sex-related developmental framing.
BR02	B	Etchell et al. (2018)	Narrative / targeted review	Childhood	Language / communication	Foundation / synthesis support	It directly synthesizes language and brain-development evidence in childhood.
BR03	B	Chaplin et al. (2013)	Meta-analysis / meta-analytic review	Childhood	Emotion / emotion recognition	Foundation / synthesis support	It is a core meta-analysis for emotion-expression phenotype in childhood.
BR04	B	Thompson et al. (2014)	Meta-analysis / meta-analytic review	NR in evidence-base note	Emotion / emotion recognition	Foundation / synthesis support	It is a core meta-analysis for emotion-recognition phenotype.
BR05	B	Margoni et al. (2023)	Meta-analysis / meta-analytic review	Infancy / early development	Social cognition / empathy / prosociality	Foundation / synthesis support	It directly evaluates early prosocial phenotype through meta-analysis.
BR06	B	Xiao et al. (2019)	Meta-analysis / meta-analytic review	Adolescence / pubertal transition	Social cognition / empathy / prosociality	Foundation / synthesis support	It meta-analyzes adolescent prosocial phenotype across multiple subtypes.

Review ID	Flow	Citation	Review type	Scope / developmental window	Primary domain	Main role in manuscript	Why retained / key contribution
BR07	B	Wood-Downie et al. (2021)	Meta-analysis / meta-analytic review	Childhood to adolescence	Language / communication	Foundation / synthesis support	It directly synthesizes sex/gender differences in social-communication phenotype in youth.
BR08	B	Edwards et al. (2024)	Meta-analysis / meta-analytic review	Adolescence / pubertal transition	Phenotypic expression	Foundation / synthesis support	It synthesizes a clinically relevant autistic phenotype with direct sex-difference implications.
BR09	B	Granocchio et al. (2023)	Narrative / targeted review	NR in evidence-base note	Language / communication	Foundation / synthesis support	It is a targeted review of sex-related differences in reading phenotype and developmental emergence.
BR10	B	McClure et al. (2000)	Meta-analysis / meta-analytic review	Infancy / early development	Emotion / emotion recognition	Foundation / synthesis support	It is a directly relevant meta-analysis of developmental sex differences in facial expression processing.
BR11	B	Leaper et al. (2004)	Meta-analysis / meta-analytic review	NR in evidence-base note	Language / communication	Foundation / synthesis support	It is a core meta-analysis of sex/gender differences in children's language phenotype.
BR12	B	Herba et al. (2004)	Narrative / targeted review	Childhood	Emotion / emotion recognition	Foundation / synthesis support	It is a focused review of the developmental emergence of facial-expression recognition.
BR13	B	Silverman et al. (2021)	Meta-analysis / meta-analytic review	Childhood	Executive function / cognitive control	Foundation / synthesis support	It is a directly relevant meta-analysis of an executive-function phenotype in early childhood.
BR14	B	Doidge et al. (2021)	Meta-analysis / meta-analytic review	NR in evidence-base note	Executive function / cognitive control	Foundation / synthesis support	It synthesizes sex differences in delay-related executive/reward decision-making across adhd.
CR01	C	Kreiser et al. (2014)	Narrative / targeted review	NR in evidence-base note	ASD clinical visibility / camouflaging	Translational / clinical visibility support	It is a key review on underrecognition and diagnostic bias in females with asd.
CR02	C	Cook et al. (2021)	Systematic review	NR in evidence-base note	ASD clinical visibility / camouflaging	Translational / clinical visibility support	It is a central systematic review on camouflaging and clinical visibility in autism.
CR03	C	Wood-Downie et al. (2021)	Review / synthesis	Childhood to adolescence	ASD clinical visibility / camouflaging	Translational / clinical visibility support	It specifically synthesizes child/adolescent evidence on sex/gender differences in camouflaging.
CR04	C	Gaub et al. (1997)	Meta-analysis / meta-analytic review	NR in evidence-base note	ADHD clinical visibility / referral and diagnosis	Translational / clinical visibility support	It is a foundational meta-analytic review on sex differences in adhd presentation.
CR05	C	Loyer et al. (2021)	Meta-analysis / meta-analytic review	NR in evidence-base note	ADHD clinical visibility / referral and diagnosis	Translational / clinical visibility support	It directly synthesizes sex differences in adhd symptoms and associated cognitive deficits.
CR06	C	Kok et al. (2016)	Narrative / targeted review	NR in evidence-base note	ADHD clinical visibility / referral and diagnosis	Translational / clinical visibility support	It reviews a female-typed adhd social phenotype with implications for lower visibility.
CR07	C	Dimitri et al. (2025)	Narrative / targeted review	Childhood to adolescence	ADHD clinical visibility / referral and diagnosis	Translational / clinical visibility support	It is a recent narrative review focused specifically on sex differences in child/adolescent adhd.

Review ID	Flow	Citation	Review type	Scope / developmental window	Primary domain	Main role in manuscript	Why retained / key contribution
CR08	C	Babinski et al. (2024)	Narrative / targeted review	NR in evidence-base note	ADHD clinical visibility / referral and diagnosis	Translational / clinical visibility support	It is a recent targeted review emphasizing identification, sex/gender issues, and future priorities in adhd research.
CR09	C	Rucklidge et al. (2010)	Narrative / targeted review	NR in evidence-base note	ADHD clinical visibility / referral and diagnosis	Translational / clinical visibility support	It is a directly relevant review of gender differences in adhd presentation and referral bias across the lifespan.
CR10	C	Yu et al. (2026)	Narrative / targeted review	NR in evidence-base note	ASD clinical visibility / camouflaging	Translational / clinical visibility support	It directly addresses under-identification of autism in females and sex-based scoring issues in a core diagnostic instrument.
CR11	C	Minutoli et al. (2026)	Narrative / targeted review	NR in evidence-base note	ASD clinical visibility / camouflaging	Translational / clinical visibility support	It is a directly relevant review focused on female underdiagnosis and misdiagnosis in autism.

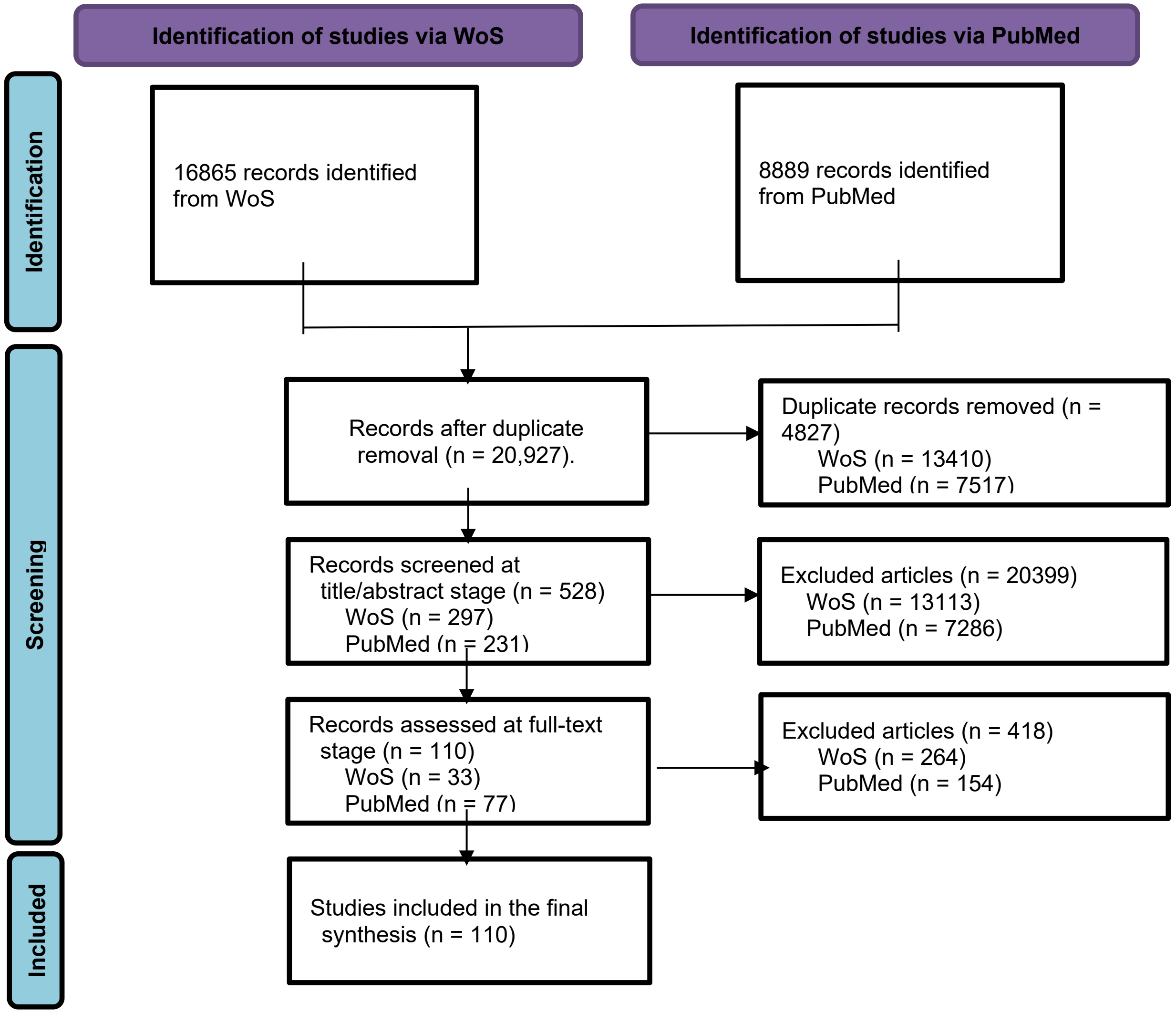

Supplementary Figure S1. Flow diagram of study identification, screening, and inclusion.
